# METTL3/YTHDC1 axis-mediated m^6^A modification of *Foxo1* mRNA promote endothelial autophagic apoptosis in diabetic atherosclerosis

**DOI:** 10.1186/s10020-026-01543-z

**Published:** 2026-07-03

**Authors:** Yujia Zhao, Longjia Tang, Hongtian Zhang, Xu Liu, Haonan Zhang, Ying Zhao, Legao Chen, Xiaoting Chen, Xiaohong Wu

**Affiliations:** 1 Geriatric Medicine Center, Key Laboratory of Endocrine Gland Diseases of Zhejiang Province, Department of Endocrinology, Zhejiang Provincial People’s Hospital, Affiliated People’s Hospital, Hangzhou Medical College, Hangzhou, Zhejiang 310014 P. R. China; 2 Department of Vascular Surgery, Zhejiang Provincial People’s Hospital, Affiliated People’s Hospital, Hangzhou Medical College, Hangzhou, Zhejiang 310014 P. R. China; 3 Department of Laboratory Medicine, Sichuan Provincial Orthopedic Hospital, Chengdu, Sichuan 610041 P. R. China

**Keywords:** Autophagy, Apoptosis, Diabetic atherosclerosis, Endothelial cells, N6-methyladenosine

## Abstract

**Background and aims:**

Endothelial dysfunction is critical in diabetic angiopathy. While our previous study established role of FOXO1 in mediating advanced glycation end products (AGEs)-induced autophagic apoptosis in human aortic endothelial cells (HAECs), the potential mechanism of N6-methyladenosine (m^6^A) modification in this process remains unknown.

**Methods:**

RNA-m^6^A methylation and methyltransferase-like 3 (METTL3) / YTH domain containing 1 (YTHDC1) expression in carotid plaques of diabetic patients and aortic plaques of diabetic apoe^−/−^ mice induced by STZ were detected by quantitative kit, immunohistochemical staining and immunofluorescence co-localization respectively. METTL3 and YTHDC1 were knocked down to observe changes in the m^6^A methylation status and expression of FOXO1. FOXO1 was overexpressed in METTL3-deficient HAECs to assess autophagic flux and apoptosis. Finally, the effect of METTL3 on atherosclerotic plaque formation was validated using endothelial cell-specific METTL3 knockout diabetic mice.

**Results:**

RNA m^6^A modification levels and the expression of the METTL3/YTHDC1 were upregulated in human diabetic carotid plaques, mouse aortic plaques, and AGEs-treated HAECs, accompanied by increased m^6^A methylation of *Foxo1* mRNA. METTL3 mediates the AGEs-induced increase in m^6^A modification of *Foxo1* mRNA, which is subsequently recognized by the YTHDC1 at a specific site in the 3’-UTR-3. This mechanism synergistically enhances FOXO1 expression and blocks autophagic flux leading to apoptosis in endothelial cells. In vivo, endothelial-specific METTL3 knockout attenuated aortic plaque formation in diabetic atherosclerotic mice and reduced FOXO1 expression along with autophagic apoptosis related proteins.

**Conclusions:**

The METTL3/YTHDC1 axis-mediated m^6^A modification of *Foxo1* mRNA promotes endothelial autophagic apoptosis in diabetic atherosclerosis, revealing a promising therapeutic target.

**Supplementary Information:**

The online version contains supplementary material available at https://doi.org/10.1186/s10020-026-01543-z.

## Introduction

Cardiovascular complications pose the primary burden of mortality in the diabetic population(Strain and Paldánius 2018). Endothelial dysfunction, induced by hyperglycemia, dyslipidemia, oxidative stress and other factors, represents an initiating trigger in the development of diabetic vascular lesions(Shi and Vanhoutte 2017). Persistent hyperglycemia drives the accumulation of advanced glycation end products (AGEs) in the bloodstream, which contributes to the pathophysiology of CVD in type 2 diabetes(Khalid et al. 2022). AGEs aggregate in the vascular wall and bind to AGE receptors (RAGE) on the surface of endothelial cells (ECs), promoting various forms of damage, such as apoptosis, shedding, etc., leading to damage to vascular structure(Yamagishi et al. 2015). Our previous research demonstrated that exposure of human aortic endothelial cells (HAECs) to AGEs impairs autophagosome-lysosome fusion, ultimately inducing autophagic apoptosis in HAECs(Zhang et al. 2019).

Forkhead box protein O1 (FOXO1), defined as a FOXO family transcription factor, plays an essential role in regulating the cell cycle, DNA repair, apoptosis, glucose metabolism, autophagy, and aging(Greer and Brunet 2005). Evidence suggests that under hyperglycemic conditions, FOXO1 activation contributes to endothelial dysfunction, thereby facilitating the development of atherosclerosis(Tanaka et al. 2009). We found that FOXO1-mediated suppression of Atg14 expression disrupts autophagosome-lysosome fusion, autophagosome accumulation, and mediating AGEs-induced autophagic apoptosis in ECs(Zhang et al. 2019). But, how AGEs/RAGE axis activates FOXO1 remains obscure.

N6-methyladenosine (m^6^A) represents the most abundant and conserved internal RNA modification in eukaryotic transcripts. Its dynamic state is precisely modulated by a tripartite system of catalytic and binding proteins, commonly termed writers, erasers, and readers(Wang and He 2014). m^6^A RNA modifications serves as a pivotal regulator of numerous cellular processes, including RNA splicing, stability, degradation, translation, cell differentiation, and metabolism(Shi et al. 2017). Dysregulation of m^6^A modifications is closely linked to the progression of cardiovascular diseases(Qin et al. 2020). For instance, disturbed blood flow and oscillatory shear stress in arterial regions promote atherosclerotic plaque formation, whereas METTL3 silencing suppresses atherosclerosis progression, highlighting a potential therapeutic target for atherosclerotic diseases(Chien et al. 2021). Under diabetic atherosclerotic conditions, whether METTL3 affects FOXO1 expression through m^6^A modification and subsequently influences endothelial function remains unknown.

In the present study, we identified a critical role for METTL3 catalyzed m⁶A modification of *Foxo1* mRNA in mediating endothelial autophagic apoptosis in diabetic atherosclerosis. We demonstrated that this modification is predominantly recognized by YTH domain containing 1 (YTHDC1) at the 3′‑UTR‑3 site of FOXO1, which in turn coordinately enhances FOXO1 expression. Collectively, our findings indicated that METTL3 promotes endothelial autophagic apoptosis in diabetic atherosclerosis through the m⁶A‑YTHDC1-dependent FOXO1 expression, revealing a novel diabetes‑specific epitranscriptomic mechanism.

## Materials and methods

### Study subjects

The discarded carotid atherosclerotic plaques were acquired as surgical specimens from patients undergoing endarterectomy procedures at Zhejiang Provincial People’s Hospital. Patient stratification into type 2 diabetic and non-diabetic cohorts was based on established diagnostic criteria, including fasting glucose levels ≥ 7.0 mmol/L, haemoglobin A1C ≥ 6.5%, or current antidiabetic medication regimen. Patient-related phenotypic informations are shown in Table S1. All patient samples in this study were obtained in accordance with the principles of the Declaration of Helsinki under informed consent, and the investigation received ethical approval from the hospital’s Institutional Review Board (2024-066).

### Cell culture and treatment

HAECs were acquired from ScienCell (ScienCell, Group Ltd) and maintained in endothelial cell medium (ScienCell, CA, USA) containing 3% fetal bovine serum (FBS). All experimental procedures were conducted using cells at passages 3 to 6 that had attained 80–90% density. Cells were exposed to 200 µg/ml AGEs-BSA (Abcam, UK) for 48 h. Gene knockdown of METTL3 and YTHDC1 was performed in HAECs by transfection with HiPerFect reagent (QIAGEN, Germany), comprising si*Mettl3*, si*Ythdc1* (RiboBio Co., Ltd. Guangzhou, China), and negative control si-NC, strictly adhering to the manufacturer’s protocol. For autophagic flux measurement, cells were treated with 100 nM Bafilomycin A1 (Baf-A1) (MedChemExpress, USA) for 4 h before harvest.

### Plasmid transfection

The FOXO1 overexpression plasmid (GeneCopoeia, China) was transfected into HAECs using Lipofectamine 3000 reagent (GLPBIO, USA) according to the manufacturer’s instructions.

### Western blotting

Protein isolation was carried out on human aortic endothelial cells, mouse aortic plaque specimens, and human carotid artery plaques employing a comprehensive lysis extraction system (KeyGEN, China). Quantitative analysis of protein content was performed with a bicinchoninic acid assay kit (Thermo Fisher Scientific, USA). For immunoblotting, 40 µg protein aliquots were electrophoresed through 8–15% SDS-PAGE, electrotransferred onto polyvinylidenefluoride (PVDF) membranes (Millipore, USA), and subsequently incubated with target-specific antibodies. Antibodies against METTL3 (ab195352), METTL14 (ab220030), WTAP (ab195380), KIAA1429 (ab271136), FTO (ab92821), ALKBH5 (ab195377), YTHDC1 (ab259990), YTHDC2 (ab220160), YTHDF1 (ab252346), YTHDF2 (ab220163), YTHDF3 (ab220161), LC3-Ⅱ (ab192890), SQSTM1(ab109012), BCL2 (ab182858), cleaved-CASP3 (ab32042), FOXO1 (ab179450), GADPH (ab181602) and ACTB (ab8227) were all from Abcam. Membrane signals were developed using an enhanced chemiluminescence (ECL) detection system (Millipore, USA) and quantified with ImageJ software version 1.37 (http://imagej.nih.gov/ij/). Protein expression levels were normalized to ACTB as an internal control, with a minimum of three biological replicates included in each experimental group.

### Immunostaining

Human carotid atherosclerotic plaques and murine aortic root specimens were processed through fixation and paraffin embedding, followed by sectioning into 5 μm slices for histological analysis. For immunohistochemistry, vascular sections were incubated with antibody targeting METTL3 (1:500) or YTHDC1 (1:500) at 4 °C overnight, followed by incubation with appropriate secondary antibodies for 1 h at room temperature. Antigen-antibody complexes were then visualized using 3,3,-diaminobenzidine, and the sections were counterstained with hematoxylin. The staining scores for the target proteins were determined by multiplying the intensity grade by the proportion of positive staining. Semi-quantitative scoring was performed by two independent pathologists blinded to the clinical characteristics, experimental groupings, and phenotypes of the subjects. The Histochemistry Score (H-score) was calculated as Σ (intensity × percentage), where intensity = 0 (negative), 1 (weak), 2 (moderate), 3 (strong), and percentage = 0-100% of positive cells (Wei et al. 2023). For immunofluorescence, frozen sections of aortic tissue from the plaque regions of mice or cells transfected for 48 h in confocal culture dishes were fixed with 4% paraformaldehyde for 15 min. The sections were permeabilized with 0.1% Triton X-100 in PBS for 10 min at room temperature and subsequently blocked with 10% normal goat serum in PBS for 1 h. Following overnight incubation at 4 °C with primary antibodies in 5% BSA, sections were washed with PBS and incubated for 2 h at room temperature with fluorophore-conjugated secondary antibodies under light-protected conditions. Samples were processed for DAPI nuclear staining (3 min) and mounted with anti-fade medium. Confocal imaging was subsequently performed to detect fluorescence signals (Olympus, Japan).

### Flow cytometric analysis of cell apoptosis

The total apoptotic cell percentage was defined as the combined percentage of cells in early apoptosis (Q3) and late apoptosis (Q2) (BD, USA). HAECs were harvested by trypsinization and subsequently stained with FITC-conjugated annexin V and PI following the manufacturer’s instructions. Apoptosis was quantified by flow cytometry (Agilent Technologies, USA), and the resulting data were subsequently analyzed with FlowJo software (Tree Star Inc).

### Quantification of m^6^A Modification

Total RNA was extracted from human carotid plaque tissues or mice aortic plaque tissues using Trizol reagent (Invitrogen, USA), which was purified using a RNA Miniprep Kit. The overall level of m^6^A in mRNA was measured using the EpiQuik m^6^A RNA Methylation Quantification Kit (EPIGENTEK, USA) according to the manufacturer’s protocol. Briefly, 200 ng of RNA and 80 µL of binding buffer were applied to the assay wells, followed by incubation with diluted capture antibody (1:1000) and detection antibody (1:2000) respectively. Finally, the absorbance was measured at 450 nm using a microplate reader (BioTek, USA) for colorimetric quantification of m^6^A content. The results were calculated based on a standard curve.

### MeRIP-seq

For MeRIP-seq analysis, 18 µg of total RNA was dispensed into 200 µL PCR tubes. Subsequently, 2 µL of 10× RNA Fragmentation Buffer was introduced to each sample, followed by complete homogenization through vortex mixing. The RNA samples were thermally equilibrated in a thermal cycler, after which 2 µL of 0.5 M EDTA solution was rapidly introduced with immediate vortex agitation. The mixture was then supplemented with 18 µL of 3 M sodium acetate buffer (pH 5.2) and homogenized, followed by introduction of 1 µL glycogen solution (20 mg/mL) with complete mixing. Finally, 600 µL of pre-chilled absolute ethanol (-20 °C) was added, and the mixture was incubated overnight at -20 °C. The precipitated product was centrifuged (12,000 g, 10 min, 30 °C), and the supernatant was discarded. The pellet was washed with 800 µL of 75% ethanol (-20 °C) and centrifuged again. The resulting pellet was dissolved in 50 µL of nuclease-free water to obtain fragmented RNA. 10% of the fragmented RNA was allocated to the input group, with the remaining material being employed to constitute the MeRIP reaction mixture. Next, 5 µg of m^6^A antibody (Synaptic Systems, USA) was incubated with magnetic beads A/G at room temperature with rotation for 30 min. The m^6^A antibody-coated beads were then combined with the MeRIP reaction solution. The complexes were washed and eluted to obtain the RNA samples. The purified RNA was subsequently used for high-throughput sequencing.

### RNA immunoprecipitation assay

To evaluate the m^6^A modification level of *Foxo1* mRNA and the interaction between FOXO1 and the reader protein YTHDC1, we performed validation through RNA immunoprecipitation (RIP) experiments, following the manufacturer’s protocol (Geneseed Biotech, China). Magnetic protein A + G beads conjugated with 5 µg of m^6^A antibody (Synaptic Systems, USA), YTHDC1 antibody (#77422, CST), or IgG antibody (#2729, CST) were added to 450 µL of cell lysate supernatant at 4 °C under rotation overnight. Subsequently, the complexes were washed and eluted to isolate the target RNA. The collected RNA was reverse-transcribed, and the relative mRNA expression levels of the target genes were detected by quantitative real-time PCR (qRT-PCR). The results were normalized to the input group.

### Luciferase reporter assay

The wild-type (WT) and mutant-type (MUT) sequences of the *Foxo1* 3’-UTR (with the GGACT motif mutated to GGCCT in the 3’-UTR) were constructed and chemically synthesized by RiboBio (Guangzhou, China), and then inserted downstream of the Renilla luciferase gene (hRluc) in the pmiR-RB-Report™ vector. The vectors were transfected into HAECs according to the manufacturer’s instructions. After 72 h of transfection, the cells were lysed, and performed using the Dual-Luciferase Reporter Assay Kit (Beyotime, China). The luminescence signals were measured using a multifunctional microplate reader (BioTek, USA). Firefly luciferase activity was used as an internal control to normalize Renilla luciferase activity.

### Animals models

*Mettl3*^fl/fl^ mice, Cdh5-CreER^T2^ mice and apoe^−/−^ mice were provided by GemPharmatech Co., Ltd (Nanjing, Jiangsu, China) and generated on a C57BL/6J strain background using CRISPR/Cas9 gene-editing technology. The mice were housed in a ventilated cage system under controlled conditions: temperature (20–26) °C, relative humidity (40–70) %, and a natural light/dark cycle. CRISPR/Cas9-mediated knockout was performed using two specific guide RNAs directed against the *Mettl3* gene: gRNA1 (forward strand, 5’-GGTTTATTGTATCATTTGAG-3’) and gRNA2 (reverse strand, 5’-CCGGTGTTTACTCTGGAGTA-3’). Tamoxifen (Sigma Aldrich, Germany) was dissolved in sterile corn oil at 10 mg/mL. Mice received intraperitoneal injections of tamoxifen at 75 mg/kg once daily for 7 consecutive days. After a 7-day recovery phase, STZ or citrate buffer injections were started. We crossed Cdh5-CreER^T2^ mice with *Mettl3*^fl/fl^ mice to generate tamoxifen-inducible endothelial-specific *Mettl3*-deficient model mice (Cdh5-CreER^T2^, *Mettl3*^fl/fl^) and the corresponding control mice. Then Cdh5-CreER^T2^- *Mettl3*^fl/fl^ mice or control mice were mated with apoe^−/−^ mice to obtain Cdh5-CreER^T2^; *Mettl3*^fl/fl^; apoe^−/−^ mice. Endothelial-specific *Mettl3* knockout was achieved in 6-week-old C57BL/6 mice through daily intraperitoneal administration of tamoxifen (75 mg/kg) over a 7-day period. Following a 7-day recovery phase, the genetically modified animals at 8 weeks of age were subjected to an atherosclerosis induction protocol to evaluate plaque formation. To eliminate the effects of sex hormones from the experiment, only male mice were used in the experiment. All experimental procedures and animal husbandry were approved by the Institutional Animal Care and Use Committee (IACUC) of GemPharmatech Co., Ltd (Ethics Number: GPTAP20230217-4, GPTAP20241120-4, GPTAP20250313-3) and were conducted in strict accordance with relevant ethical guidelines. Our study complies with the Guide for the Care and Use of Laboratory Animals published by the US National Institutes of Health (NIH Publication, 8th edition, 2011).

### Mouse diabetic atherosclerosis induction and lesion analysis

Four experimental groups were included: (1) Control: apoe^⁻/⁻^ mice injected with citrate buffer; (2) Diabetic atherosclerosis: apoe^⁻/⁻^ mice injected with STZ (55 mg/kg/day for 5 d); (3) EC *Mettl3*^⁺/⁺^: Cdh5-CreER^T2^; *Mettl3*ᶠˡ/ᶠˡ; apoe^⁻/⁻^ mice receiving corn oil followed by STZ and (4) EC *Mettl3*^fl/fl^: Cdh5-CreER^T2^; *Mettl3*ᶠˡ/ᶠˡ; apoe^⁻/⁻^ mice receiving tamoxifen (75 mg/kg/day for 7 d) followed by STZ. Both groups of mice were fed a standard diet until 20 weeks of age. Mice were anaesthetized via 1% isoflurane inhalation for collecting blood. Fasting blood glucose (FBG) was measured after a 6 h fast on day 3 and day 7 after the final STZ injection. Mice were considered diabetic if FBG ≥ 11.1 mmol/L at both time points. In the STZ-treated groups (apoe^⁻/⁻^+STZ group, EC *Mettl3*^+/+^ group and EC *Mettl3*^fl/fl^ group), 20 out of 22mice (90.9%) met the diabetic criteria and were included in the final analysis. Control apoe^⁻/⁻^ mice maintained FBG below 11.1 mmol/L throughout the experiment.

Euthanasia of mice by CO_2_, using a gradual-fill, controlled-flow method, with the displacement rate maintained at 30%–70% of the chamber volume per minute. The aorta (from ascending to iliac bifurcation) was isolated, fixed in 4% paraformaldehyde, and stained with 0.5% Oil Red O (60% isopropanol) for 60 min at room temperature. En face plaque area was quantified using Image J and expressed as percentage of total aortic area. For cross-sectional analysis, 8 μm cryosections of the aortic root were stained with H&E or Oil Red O. Plaque burden was calculated as (lesion area / vessel area) × 100% (≥ 3 sections/mouse, 80 μm apart). All quantifications were performed blindly by two independent investigators.

### Statistical analysis

All experimental results are expressed as mean values ± standard deviation. GraphPad Prism (v8.0) was utilized for statistical computations. Dataset normality was verified through Shapiro-Wilk testing, with variance homogeneity confirmed by Brown-Forsythe analysis. Intergroup comparisons were conducted using Student’s *t*-test assuming equal variances. Multiple group comparisons were conducted using one-way or two-way ANOVA with Tukey’s honestly significant difference test for post hoc analysis. All experiments were independently repeated at least three times. Significance levels were defined as follows: ^****^P < 0.0001, ^***^P < 0.001, ^**^P < 0.01, ^*^P < 0.05.

## Results

### Increased RNA-m^6^A modification in diabetic vascular plaques and Identification of *Foxo1* as a key m⁶A target in AGEs-treated HAECs

**Fig. 1:**
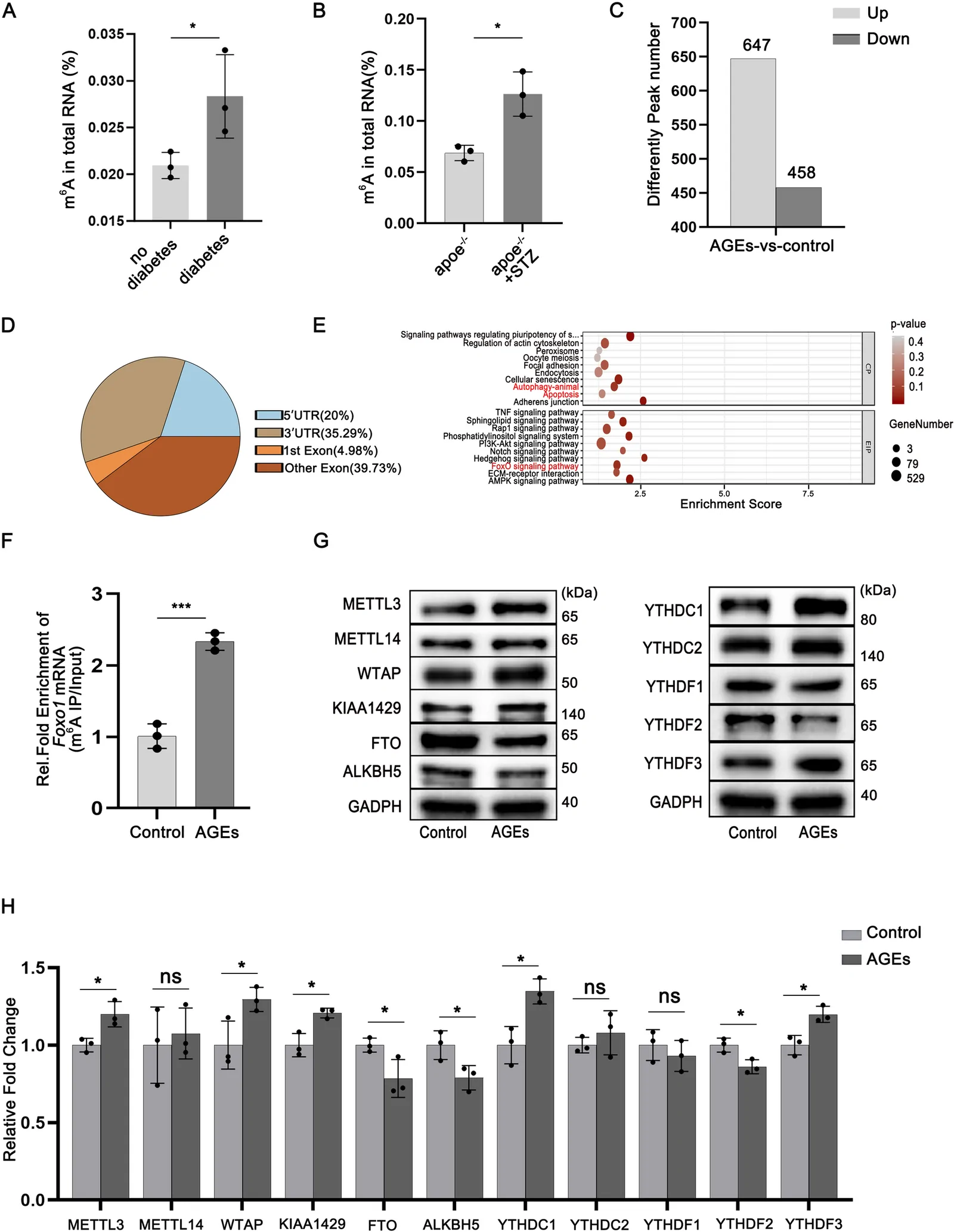
Increased RNA-m^6^A modification in diabetic vascular plaques and Identification of *Foxo1* as a key m⁶A target in AGEs-treated HAECs. The m^6^A modification levels in carotid plaques from diabetic patients (**A**) and aortic plaques of STZ-treated apoe^−/−^ mice (**B**) was quantified with using the EpiQuik™ m^6^A RNA Methylation Quantification Kit. *n* = 3. **C** Quantification of differentially methylated m^6^A peaks between AGEs-treated and control groups. **D** A pie chart illustrating the distribution of differential m^6^A peaks across various transcript regions. **E** Pathway enrichment analysis of the genes associated with differential peaks was performed using the Kyoto Encyclopedia of Genes and Genomes (KEGG) database, and the significance of gene enrichment in each pathway was assessed using the hypergeometric distribution test. In the figure, the X-axis represents the enrichment score. Bubbles with larger sizes correspond to pathways containing a greater number of differentially expressed genes. The bubble color transitions from gray to red, indicating progressively smaller p-values and thus increasing statistical significance. **F** RIP assay demonstrating the interaction between m^6^A and *Foxo1* mRNA in control and AGEs-treated group. *Foxo1* mRNA was enriched using an m6A-specific antibody and its abundance was quantified via qRT-PCR. *n* = 3. **G** and **H** Western blot analysis showing changes in the expression of m^6^A-related “writer” proteins (METTL3, METTL14, WTAP and KIAA1429), “eraser” proteins (ALKBH5 and FTO), and “reader” proteins (YTHDC1-2 and YTHDF1-3) in AGEs-treated HAECs. *n* = 3. Values represent mean ± SD, and analyzed by Student’s *t*-test in (**A**, **B**, **F** and **H**). *n* = 3 independent biological replicates. ^*^P < 0.05, ^**^P < 0.01, ^***^P < 0.001, ^****^P < 0.0001

To investigate changes in m^6^A modification levels in the context of diabetic atherosclerosis, we analyzed carotid plaques from diabetic patients following carotid plaque endarterectomy. RNA-m^6^A methylation quantitative assays revealed that, compared to non-diabetic patients, those with diabetes exhibited significantly higher RNA-m^6^A modification levels in their carotid plaques (Fig. 1A, S1 and Table S2) Consistent results were observed in the aortic plaques of diabetic atherosclerotic mice model (Fig. 1B).

To further determine the m^6^A modification profiles in AGEs-treated HAECs, we performed high-throughput RNA sequencing (MeRIP-seq) using an antibody-based method to recognize m^6^A modifications. Results showed that 1,105 significantly altered peaks in AGEs-treated samples compared with controls (P < 0.05, log_2_ FC > 0.58), with 647 peaks upregulated and 458 peaks downregulated (Fig. 1C). Notably, most of the significantly altered peaks in the AGEs-treated group were located in other exons (39.73%), with the remaining distributed in the 3’ UTR (35.29%), 5’ UTR (20%), and the first exon (4.98%) (Fig. 1D). Our previous work established the pivotal role of autophagic apoptosis imbalance and FOXO signaling in diabetic endothelial dysfunction(Zhang et al. 2019). Pathway enrichment analysis of the genes associated with differential peaks was performed using the Kyoto Encyclopedia of Genes and Genomes (KEGG) database, and the significance of gene enrichment in each pathway was assessed using the hypergeometric distribution test. Analysis of KEGG pathways demonstrated an enrichment of highly methylated mRNAs in autophagy and FOXO signaling pathways (Fig. 1E). Since FOXO1 is a master transcription factor, we performed a MeRIP-qPCR assay, which demonstrated a dramatic and specific increase in *Foxo1* mRNA-m^6^A levels in AGEs-treated HAECs relative to controls (Fig. 1F).

To investigate the dynamic changes in the m^6^A regulatory network, we examined the expression of m^6^A regulators. Western blot assay revealed differential expression of key m^6^A regulators such as METTL3, WTAP, KIAA1429, YTHDC1, and YTHDF3 was significantly upregulated, while FTO, ALKBH5 and YTHDF2 expressions were notably downregulated, with no changes in METTL14, YTHDF1 and YTHDC2 (Fig. 1G and H).

### Increased expression of METTL3 in diabetic vascular plaques

**Fig. 2:**
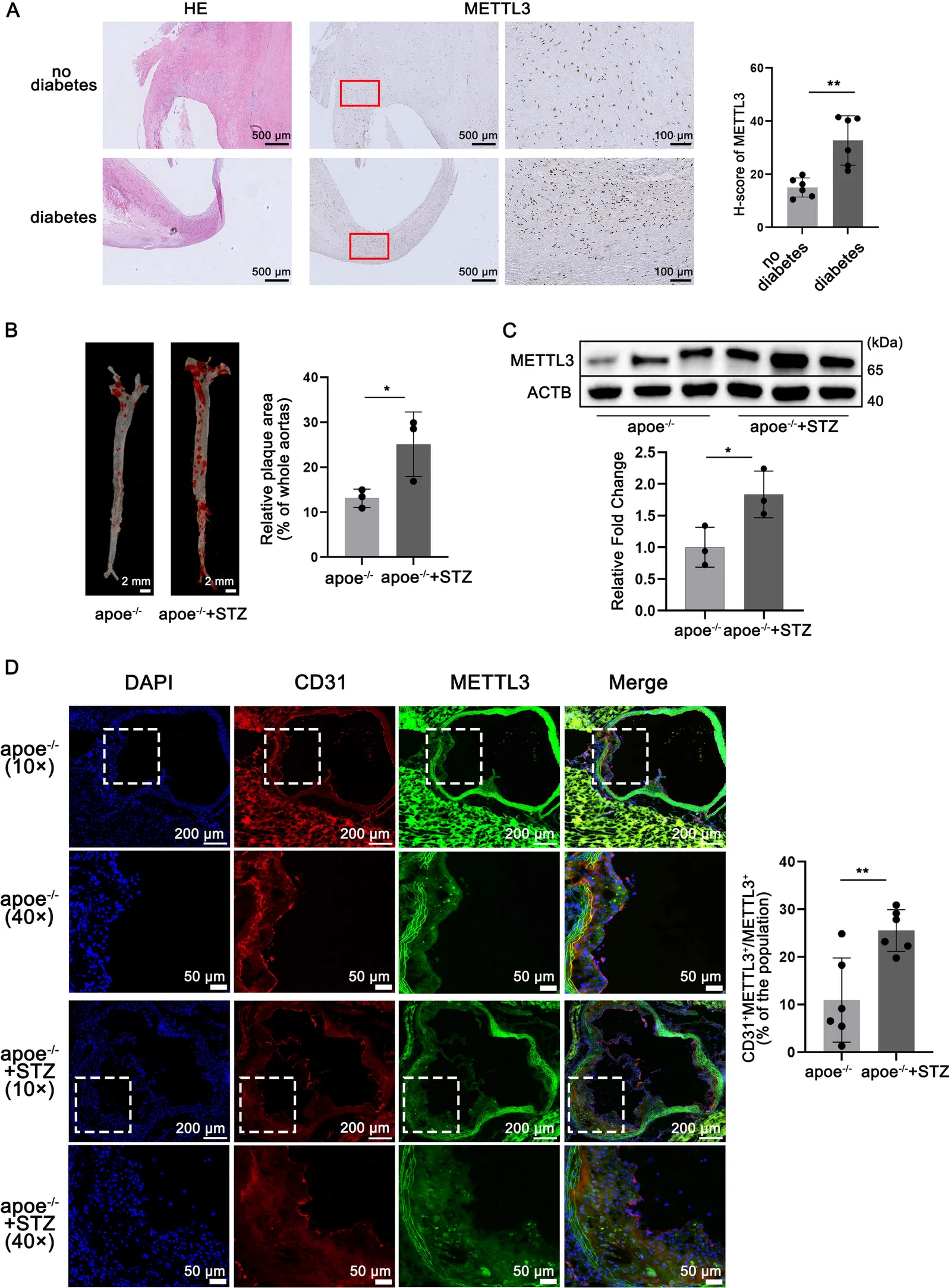
Elevated METTL3 expression in diabetic vascular plaques. **A** Representative images and scores of METTL3 IHC staining in carotid plaques from diabetic patients. Scale bar = 500, 100 μm. *n* = 6. **B** Representative en face Oil Red O staining images of whole aortas and quantitative analysis showing increased plaque formation in apoe^−/−^+STZ mice compared to controls. Scale bar = 2 mm. *n* = 3. **C** Representative immunoblot showing METTL3 protein levels in aortic plaques from STZ-induced diabetic apoe^−/−^ mice. *n* = 3. **D** Immunofluorescence staining and quantitative analysis demonstrated the co-localization of METTL3 with CD31 in mouse aortic plaque regions. Scale bar = 200, 50 μm. *n* = 6. Data are presented as mean ± SD, and analyzed by Student’s *t*-test in all. *n* = 6 (**A**), *n* = 3 (**B**, **C**), *n* = 6 (**D**) independent biological replicates. ^*^P < 0.05, ^**^P < 0.01, ^***^P < 0.001, ^****^P < 0.0001

To investigate whether METTL3 is involved in diabetic atherosclerosis, we collected carotid plaques from diabetic patients and aortic plaques of diabetic atherosclerotic mice. Immunohistochemical staining revealed that, compared to non-diabetic patients, METTL3 expression was significantly elevated in carotid plaques of diabetic patients (Fig. 2A). To establish a murine model of diabetic atherosclerosis, apoe^⁻/⁻^ mice were subjected to intraperitoneal administration of STZ and maintained on a standard diet until 20 weeks of age. Compared to the non-diabetic atherosclerosis group, mice with superimposed diabetes exhibited significant reductions in body weight, alongside pronounced elevations in both blood glucose and lipid parameters (Figure S2A-E and Table S3). Notably, analysis of en face Oil Red O staining of whole aortas revealed an increase in plaque area within the diabetic cohort relative to controls (Fig. 2B and Table S4). Furthermore, histopathological examination of aortic root sections demonstrated a concomitant significant increase in intra-plaque lipid deposition and necrotic core area in diabetic atherosclerosis mice compared to their non-diabetic counterparts (Figure S2F). Immunoblotting revealed elevated METTL3 protein levels in the aortic plaque regions of diabetic atherosclerotic mice compared with the atherosclerosis-only group (Fig. 2C). Additionally, immunofluorescence staining showed that, compared to atherosclerotic mice, the co-localization of METTL3 with CD31 was significantly increased in the aortic plaque area of diabetic atherosclerotic mice, indicating elevated METTL3 expression in endothelial cells (Fig. 2D). These findings, along with in vitro experiments, suggest that METTL3 expression was increased in diabetic vascular plaques, especially in endothelial cells.

### METTL3 participates in AGEs-induced autophagic apoptosis of HAECs by regulating m^6^A modification and protein expression of FOXO1

**Fig. 3:**
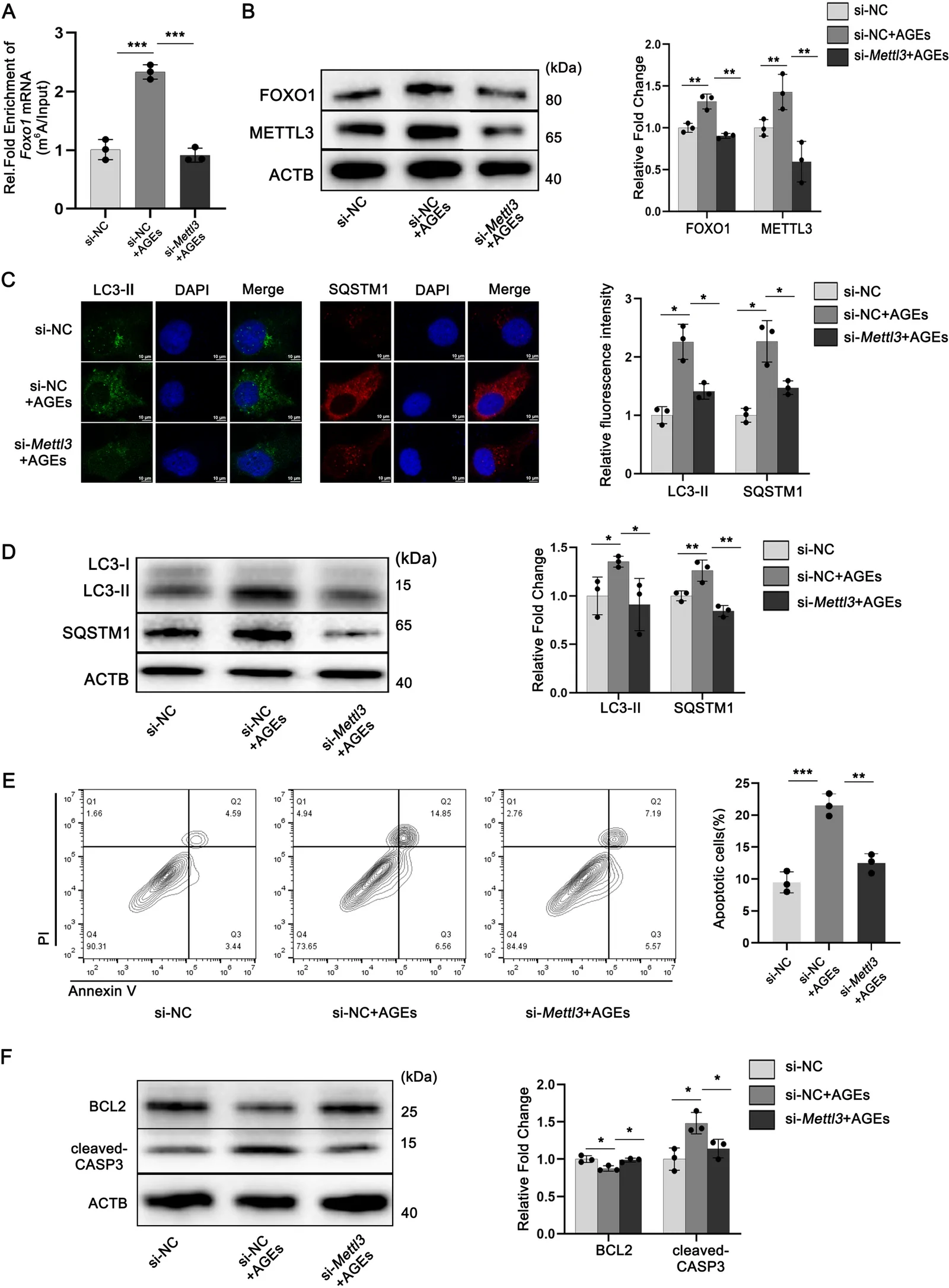
METTL3 regulates FOXO1 m^6^A modification and protein expression, contributing to AGEs-induced autophagic apoptosis in HAECs. HAECs were subjected to transfection using si*Mettl3* or control siRNA for 24 h, followed by AGEs treatment for 48 h. RIP assay and western blot analysis showing that knockdown of METTL3 reversed the elevated m^6^A modification levels of *Foxo1* mRNA (**A**) and FOXO1 protein expression (**B**) induced by AGEs. *n* = 3. Confocal microscopy images (**C**) and western blot analysis (**D**) indicating that knockdown of METTL3 reversed the AGEs-induced increases in LC3-Ⅱ and SQSTM1 expression. Scale bar = 10 μm. *n* = 3. **E** Flow cytometry analysis demonstrated that knockdown of METTL3 reversed the AGEs-induced apoptosis levels. *n* = 3. **F** Western blot analysis demonstrated that METTL3 knockdown rescued the AGEs-mediated downregulation of BCL2 and attenuated the elevation of cleaved-CASP3. *n* = 3. Data are presented as mean ± SD, and analyzed by one-way ANOVA with Tukey’s post hoc test in (**A**, **B**, **C**, **D**, **E** and **F**). *n* = 3 independent biological replicates. ^*^P < 0.05, ^**^P < 0.01, ^***^P < 0.001, ^****^P < 0.0001

Since METTL3 expression and m^6^A modification levels of *Foxo1* mRNA are significantly elevated in AGEs-treated HAECs, and FOXO1 participates in AGEs-induced autophagic apoptosis of HAECs(Zhang et al. 2019), we speculated that METTL3 might be involved in AGEs-induced autophagic apoptosis of HAECs. We knocked down the METTL3 expression by si*Mettl3*, finding that si*Mettl3* significantly reversed the elevated m^6^A modification levels of *Foxo1* mRNA and FOXO1 protein expression induced by AGEs (Fig. 3A and B). Immunofluorescence (Fig. 3C) and western blot (Fig. 3D) results showed that *siMettl3* significantly reversed the AGEs‑induced alterations in the autophagy‑related markers LC3‑II and SQSTM1. To determine whether autophagic flux is blocked, we performed a Baf-A1 (100 nM, 4 h) chase assay (Figure S3A–B). Compared with the control group, Baf-A1 increased the levels of LC3‑II and SQSTM1, indicating that autophagic flux was blocked. AGEs alone caused marked accumulation of both markers, which was not further enhanced by Baf-A1, confirming severe impairment of late-stage autophagic degradation. Importantly, METTL3 knockdown reduced the AGEs-induced levels of LC3-II and SQSTM1, and both markers were further increased upon Baf-A1 exposure. Collectively, these data demonstrate that METTL3 silencing alleviates AGEs-induced autophagic flux blockade in HAECs. Flow cytometry and western blot results confirmed that si*Mettl3* significantly reversed the AGEs-induced apoptosis, as well as the reduction in BCL2 and the increase in cleaved-CASP3 (caspase 3) (Fig. 3E and F).

Meanwhile, to determine the temporal sequence of *Foxo1* m⁶A modification, autophagy, and apoptosis, HAECs were treated with AGEs for 0, 12, 24, and 48 h (Figure S4A–D). At 12 h, *Foxo1* m⁶A levels progressively increased. At 24 h, FOXO1 protein and LC3‑II were elevated, whereas SQSTM1 and cleaved-CASP3 remained low. At 48 h, m⁶A peaked, SQSTM1 massively accumulated, LC3‑II stayed elevated, BCL2 dropped, and cleaved-CASP3 was activated, indicating a transition from autophagic flux blockade to apoptosis. These results suggest that METTL3 may regulate AGEs‑induced autophagic apoptosis via FOXO1 modification and protein expression.

**Fig. 4:**
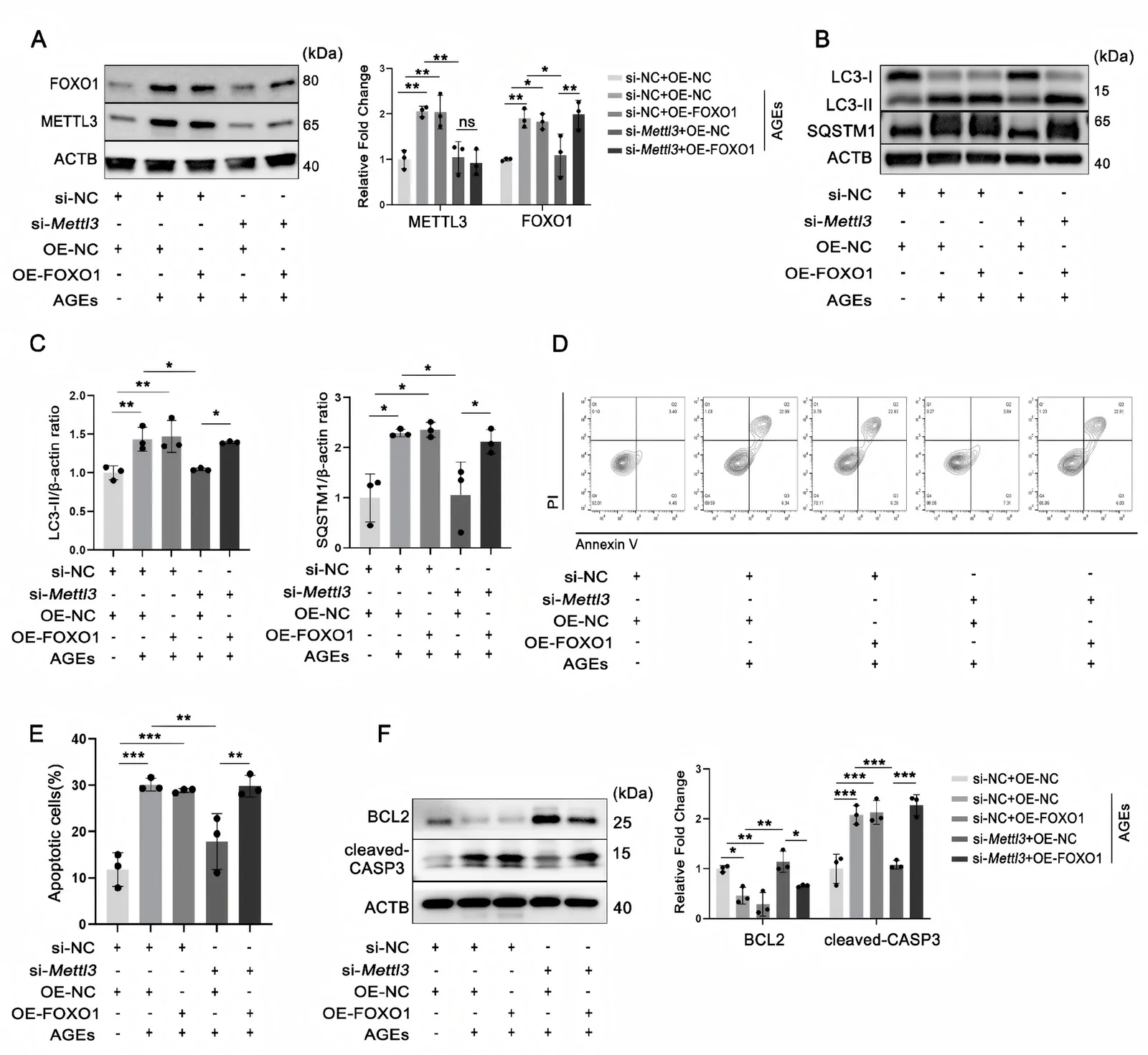
METTL3 governs AGEs-induced endothelial autophagic apoptosis via an essential downstream effector FOXO1. HAECs were first transfected with si*Mettl3* or control siRNA for 24 h. After medium replacement, cells were then transfected with FOXO1 overexpression plasmid or control vector (NC) for 4–6 h, followed by another medium change and subsequent treatment with AGEs for 48 h. **A** Western blot analysis and quantification of METTL3 and FOXO1 protein expression after METTL3 knockdown and FOXO1 overexpression. *n* = 3. **B**–**C** Western blot analysis and quantification of autophagy‑related proteins LC3‑II and SQSTM1 under the same conditions. *n* = 3. **D**–**E** Flow cytometric analysis and quantification of apoptosis levels. *n* = 3. **F** Western blot analysis and quantification of apoptosis‑related proteins BCL2 and cleaved‑CASP3. *n* = 3. All data are presented as mean ± SD and were analyzed by one‑way ANOVA followed by Tukey’s post hoc test in (**A**, **C**, **E** and **F**). *n* = 3 independent biological replicates. ^*^P < 0.05, ^**^P < 0.01, ^***^P < 0.001, ^****^P < 0.0001, ns, not significant

To determine whether FOXO1 is the key downstream effector of METTL3, we performed rescue experiments. HAECs were co‑transfected with siMettl3 or control siRNA together with FOXO1 overexpression (OE‑FOXO1) or empty vector, followed by AGEs treatment (Fig. 4A). METTL3 knockdown alleviated AGEs‑induced endothelial injury, as evidenced by reduced LC3‑II and SQSTM1 accumulation, decreased apoptosis, restored BCL2, and lowered cleaved‑CASP3 (Fig. 4B–F). Notably, FOXO1 overexpression reversed these protective effects, restoring autophagic blockade and apoptosis to levels similar to those in AGEs‑only treated cells. These results demonstrate that FOXO1 acts as a critical downstream mediator of METTL3‑induced endothelial dysfunction.

### YTHDC1 recognizes m^6^A modification sites in the 3’-UTR of *Foxo1* mRNA and regulates FOXO1 protein expression

**Fig. 5:**
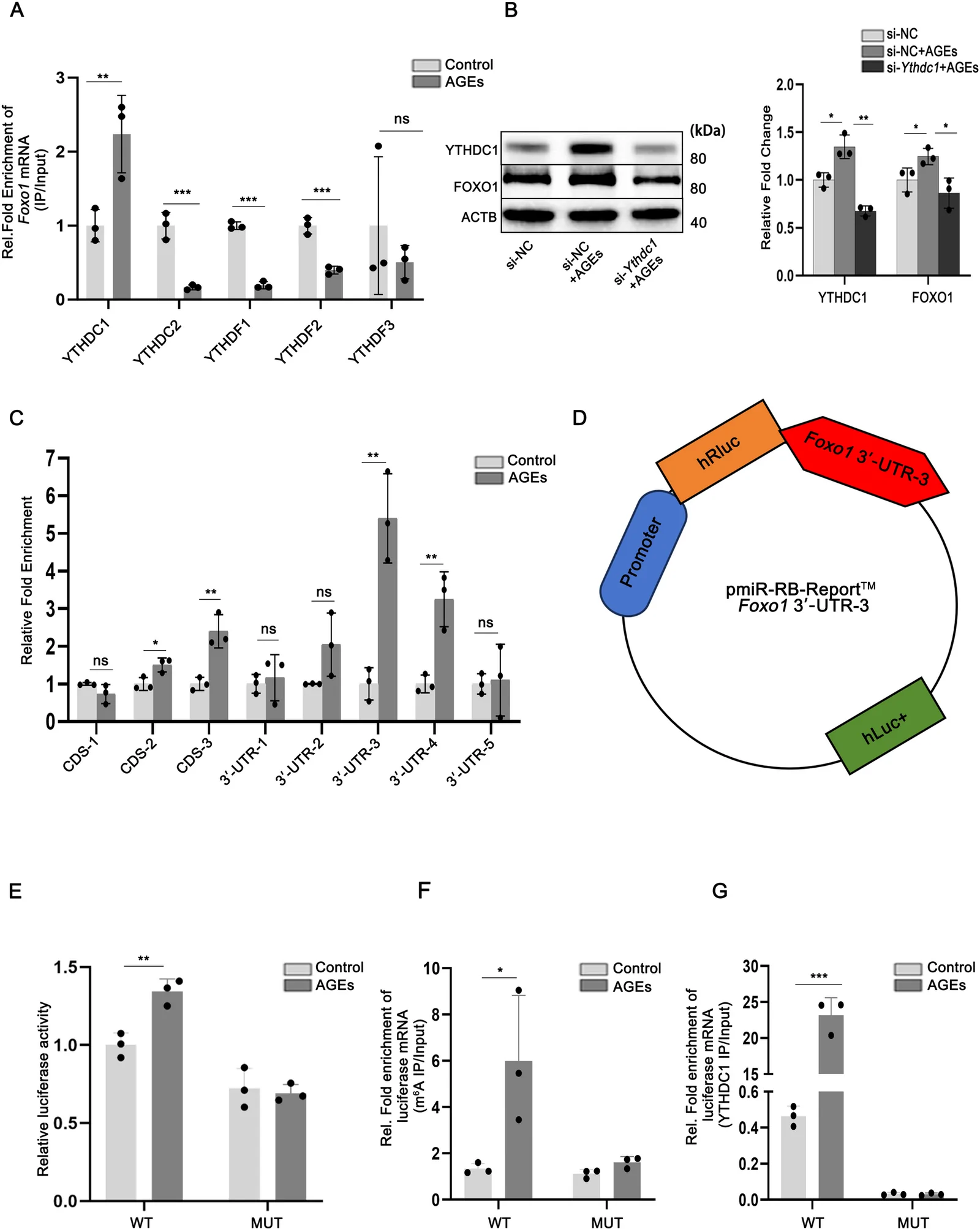
YTHDC1 recognizes m^6^A sites in the 3′-UTR of *Foxo1* mRNA and regulates FOXO1 protein expression in HAECs. **A** RIP assay analyzing the interaction between *Foxo1* mRNA and various m^6^A “reader” proteins. *n* = 3. **B** HAECs underwent a 24 h transfection period with either si*Ythdc1* or non-targeting control siRNA, followed by 48 h exposure to AGEs. Western blot analysis showing that knockdown of YTHDC1 reversed AGEs-induced upregulation of FOXO1 expression. *n* = 3. **C** RIP assay detecting the interaction between m^6^A and putative m^6^A modification sites on *Foxo1* mRNA. *n* = 3. **D** To validate these findings, we constructed pMIR-REPORT plasmids containing either wild-type or mutated modification sites. **E** Luciferase reporter assays revealed that AGEs stimulation increased luciferase activity in the wild-type (3’-UTR-3-WT) plasmid, with no effect on the mutated (3’-UTR-3-MUT) plasmid. *n* = 3. RIP assays demonstrated that AGEs stimulation enhanced m^6^A modification (**F**) and YTHDC1 binding to the *Foxo1* 3′-UTR-3 region (**G**) in the wild-type plasmid with no effect on the mutant plasmid. *n* = 3. Data are presented as mean ± SD, and analyzed by Student’s *t*-test in (**A**, **C**, **E**, **F** and **G**) or one-way ANOVA with Tukey’s post hoc test in (**B**). *n* = 3 independent biological replicates. ^*^P < 0.05, ^**^P < 0.01, ^***^P < 0.001, ^****^P < 0.0001

After m^6^A modifications are “written” onto mRNA by methyltransferases, specific reader proteins such as YTHDC1/2 and YTHDF1/2/3 must “read” this modification to interpret its biological significance(Yang et al. 2018). To investigate the interactions between these reader proteins and *Foxo1* mRNA, we performed RIP assays using antibodies against each reader protein to enrich *Foxo1* mRNA, followed by qRT-PCR to measure *Foxo1* mRNA abundance. The results revealed that AGEs markedly increased the specific binding of YTHDC1 to *Foxo1* mRNA (Fig. 5A and Figure S5A-E). Knockdown of YTHDC1 significantly reversed the AGEs-induced upregulation of FOXO1 expression (Fig. 5B). To further explore which site YTHDC1 directly interacts with *Foxo1* mRNA, we used the SRAMP database to predict the m^6^A modification sites in *Foxo1* mRNA, identifying eight highly reliable sites, three of which were located in the CDS region, and five in the 3’-UTR region. We designed primers targeting these eight modification sites and performed RIP assays using an m^6^A antibody to enrich the modified regions, followed by qRT-PCR to assess the abundance of each suspected modification site. The results showed that AGEs significantly promoted m^6^A modification at the 3’-UTR-3 site (Fig. 5C). To validate these findings, we generated pMIR-REPORT plasmids harboring either wild-type or mutated modification sites (Fig. 5D). Functional assessment using luciferase reporters revealed that AGEs stimulation increased luciferase activity in the wild-type (3’-UTR-3-WT) plasmid, with no effect on the mutated (3’-UTR-3-MUT) plasmid (Fig. 5E). RIP assays further demonstrated that AGEs stimulation enhanced m^6^A modification and YTHDC1 binding to the *Foxo1* 3′-UTR-3 region in the wild-type plasmid, whereas the mutant plasmid was rendered insensitive to this induction (Fig. 5F and G). Therefore, our findings indicate that YTHDC1 recognizes the m^6^A modification site within the 3′-UTR-3 of *Foxo1* mRNA and regulates FOXO1 protein expression.

### Increased expression of YTHDC1 in diabetic vascular plaques

**Fig. 6:**
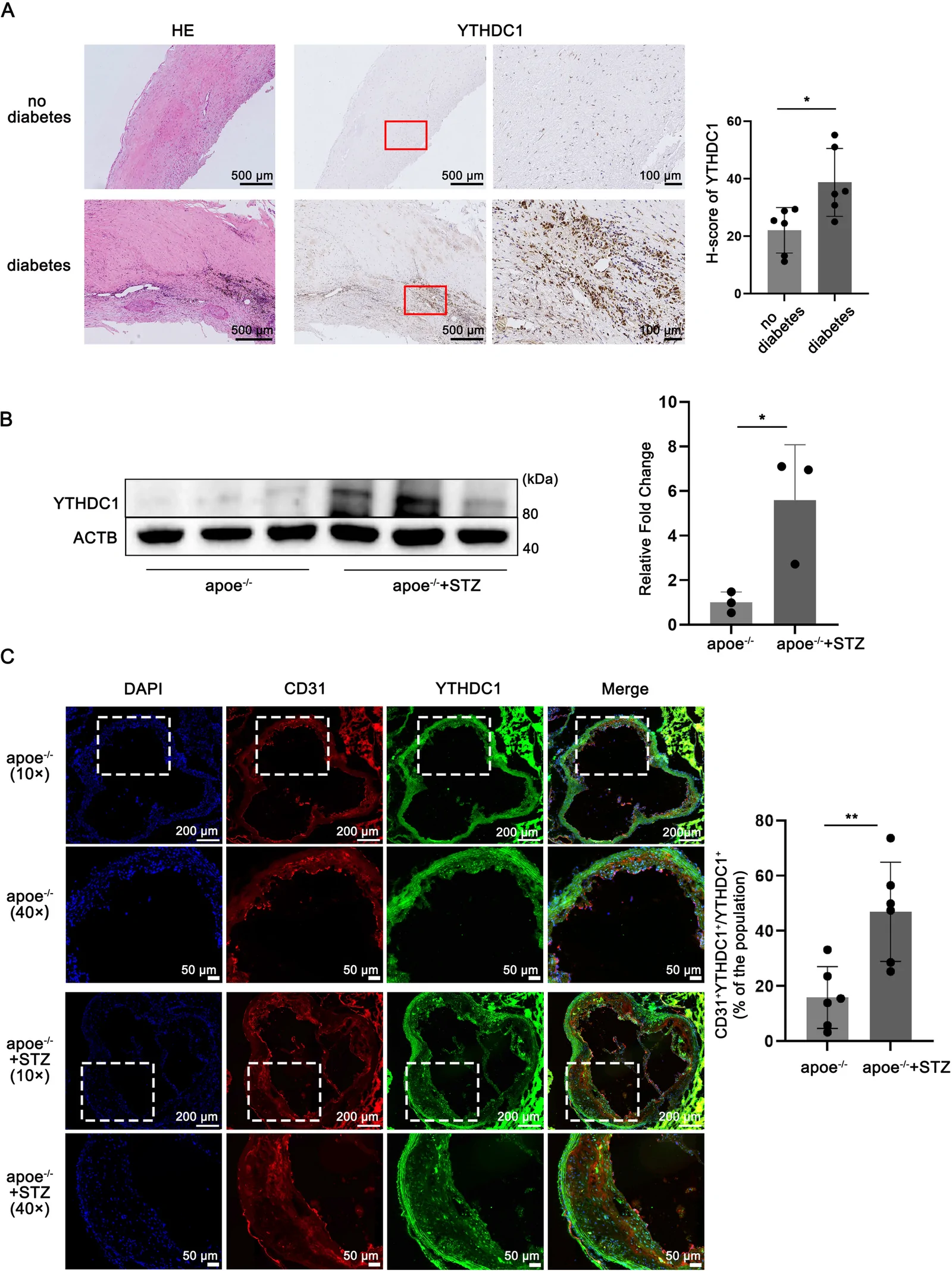
Elevated YTHDC1 expression in diabetic vascular plaques. **A** Representative images and scores of YTHDC1 IHC staining in carotid plaques from diabetic patients. Scale bar = 500,100 μm. *n* = 6. **B** Western blot analysis was performed to detect YTHDC1 expression in aortic plaques of STZ-treated apoe^−/−^ mice. *n* = 3. **C** Immunofluorescence staining and quantitative analysis demonstrated the co-localization of YTHDC1 with CD31 in mouse aortic plaque regions. Scale bar = 200, 50 μm. *n* = 6. Values represent mean ± SD, and analyzed by Student’s *t*-test in all. *n* = 6 (**A**), *n* = 3 (**B**), *n* = 6 (**C**) independent biological replicates. ^*^P < 0.05, ^**^P < 0.01, ^***^P < 0.001, ^****^P < 0.0001

Previous investigations have established that changes in YTHDC1 expression are closely linked to atherosclerosis development(Zhao et al. 2024). To investigate whether YTHDC1 is changed in diabetic atherosclerosis, we collected carotid plaques from diabetic patients and aortic plaques of diabetic atherosclerotic mice. Immunohistochemical staining revealed that, compared to non-diabetic patients, YTHDC1 expression was significantly elevated in carotid plaques of diabetic atherosclerotic patients (Fig. 6A). Immunoblotting analysis revealed markedly elevated YTHDC1 protein levels in aortic plaque specimens from diabetic atherosclerotic mice relative to atherosclerosis mice (Fig. 6B). Additionally, immunofluorescence staining showed that, compared to atherosclerotic mice, the co-localization of YTHDC1 with CD31 was notably higher in the aortic plaque area of diabetic atherosclerotic mice, indicating elevated YTHDC1 expression in endothelial cells (Fig. 6C). These findings confirm that YTHDC1 expression was increased in diabetic vascular plaques, including endothelial cells.

### Endothelial-specific METTL3 knockout attenuates atherosclerosis in STZ-treated apoe^−/−^ mice

**Fig. 7:**
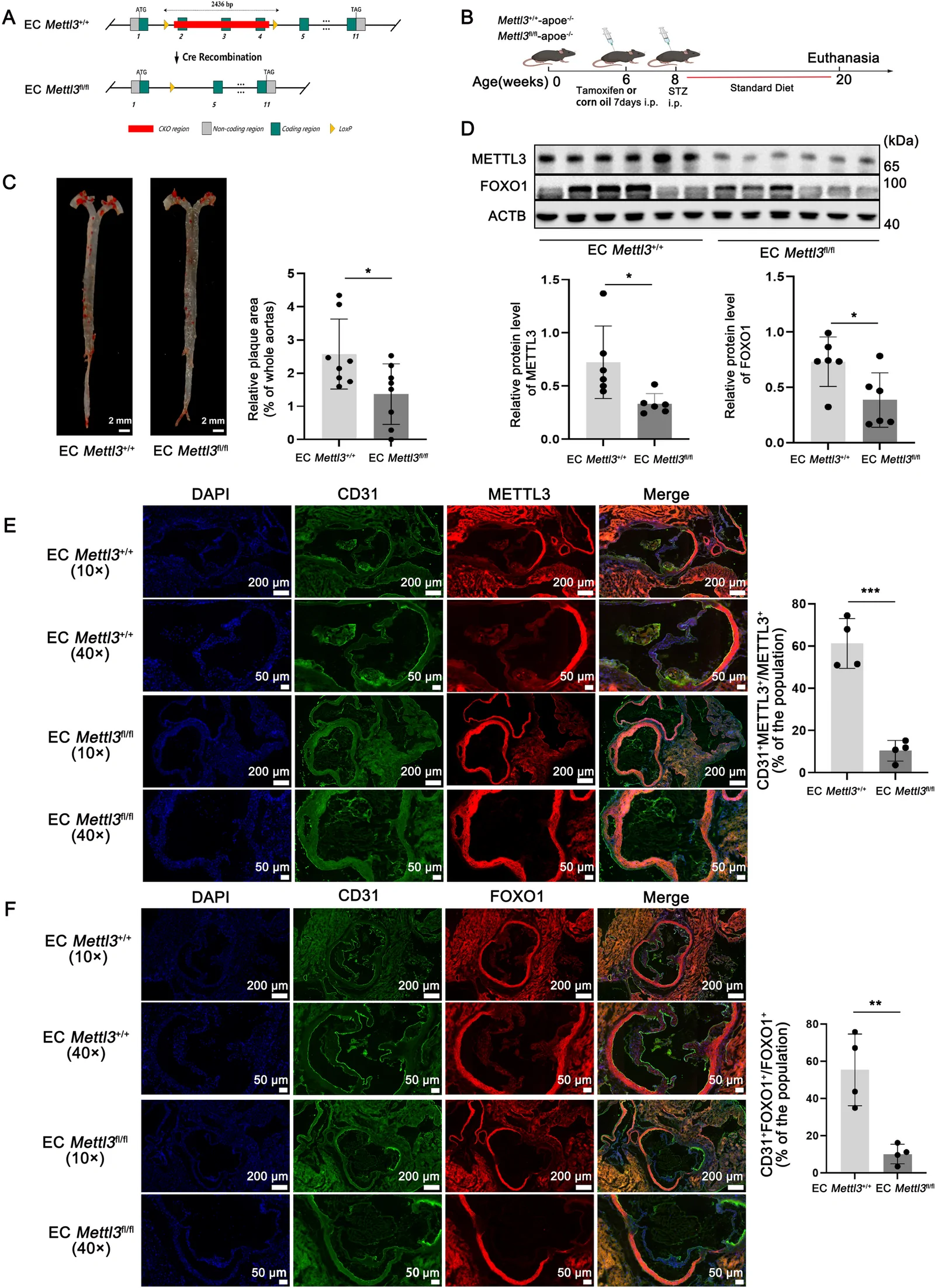
Endothelial cell-specific knockout of METTL3 significantly ameliorates atherosclerotic plaque formation in diabetic mice. **A** Strategy for generating endothelial cell-specific METTL3 knockout mice. **B** Schematic of diabetic atherosclerosis induction in EC *Mettl3*^+/+^-apoe^−/−^and EC *Mettl3*^fl/fl^-apoe^−/−^ mice. Corn oil or Tamoxifen (75 mg/kg/day for 7 days) was administered, followed by a 7-day recovery phase and then streptozotocin (STZ) injection. After 5 days, mice were maintained on a standard diet until 20 weeks. **C** Representative en face Oil Red O staining images of whole aortas and quantitative analysis showing reduced plaque formation in EC *Mettl3*^fl/fl^ mice compared to controls. Scale bar = 2 mm. *n* = 8. **D** Western blot analysis showing METTL3 and FOXO1 protein levels in aortic specimens, with corresponding quantification. *n* = 6. Immunofluorescence staining and quantitative analysis of METTL3 (**E**) and FOXO1 (**F**) in aortic plaque, with co-localization analysis of CD31 (an EC marker), METTL3, or FOXO1. Scale bar = 200, 50 μm. *n* = 4. Data are presented as mean ± SD, and analyzed by Student’s *t*-test in (**C**, **D**, **E** and **F**). *n* = 8 (**C**), *n* = 6 (**D**), *n* = 4 (**E**, **F**) independent biological replicates. ^*^P < 0.05, ^**^P < 0.01, ^***^P < 0.001, ^****^P < 0.0001

To investigate the effect of EC-specific METTL3 knockout on atherosclerotic plaque formation in diabetic mice, tamoxifen-inducible EC-specific METTL3 knockout mice were created. Based on *Mettl3* gene information from the NCBI database, we inserted loxP sites flanking exons 2–4 of the *Mettl3*-201 transcript to achieve conditional knockout (Fig. 7A). By crossing Cdh5-P2A-CreERT2 mice with *Mettl3*^fl/fl^ and apoe^−/−^ mice followed by 7-day tamoxifen induction, we successfully obtained EC-specific METTL3 knockout mice. The genotyping of the EC *Mettl3*^fl/fl^ mice was confirmed through agarose gel electrophoresis of the PCR fragments in the CRISPR -targeting region amplified from genomic DNA isolated from mouse tails (Figure S6A). Endothelial-specific METTL3 knockout mice received daily intraperitoneal administration of STZ (55 mg/kg/day for five consecutive days) to induce diabetic atherosclerotic conditions and maintained on a standard diet for 20 weeks (Fig. 7B). No significant differences in body weight or blood glucose levels were observed between groups, indicating that EC-specific METTL3 knockout did not significantly affect these parameters(Figure S6B and S6C, Table S5). En face Oil Red O staining of aortas revealed a significant reduction in atherosclerotic plaque formation in EC *Mettl3*^fl/fl^ mice compared with controls (Fig. 7C). Protein analysis of vascular tissues demonstrated markedly decreased expression of both METTL3 and FOXO1 in EC *Mettl3*^fl/fl^ mice (Fig. 7D), with upregulated expression of BCL2 and decreased expression of cleaved-CASP3 (caspase3), SQSTM1 and LC3-Ⅱ (Figure S7A and S7B). Immunofluorescence co-localization staining further confirmed METTL3 and FOXO1 in endothelial cells was significantly lower in the atherosclerotic plaque area of EC *Mettl3*^fl/fl^ mice (Fig. 7E and F). These results collectively demonstrate that EC-specific METTL3 knockout ameliorated atherosclerotic plaque formation in diabetic mice, indicating that METTL3 likely promotes plaque formation through regulation of FOXO1 expression.

## Discussion

**Fig. 8:**
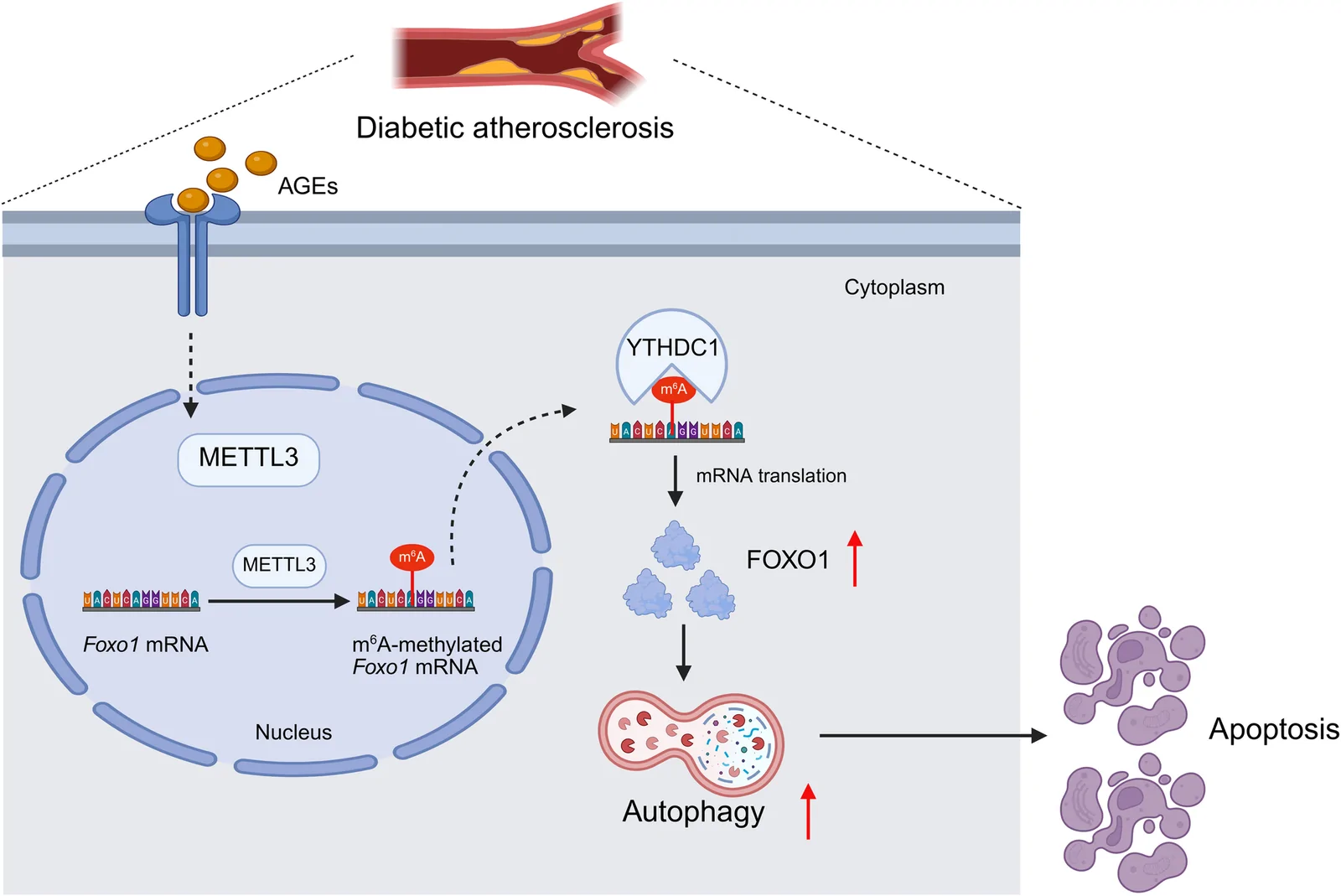
This schematic illustrates the molecular mechanism by which the METTL3/YTHDC1 axis promotes autophagic apoptosis of endothelial cells in diabetic atherosclerosis. The chronic accumulation of AGEs significantly enhances the METTL3-mediated m^6^A modification of *Foxo1* mRNA, further recognized by a YTHDC1-dependent manner, resulting in the upregulation of FOXO1 expression, leading to autophagic apoptosis and aggravated diabetic atherosclerosis progression

In this study, we first observed that the m^6^A modification levels were significantly altered in diabetic vascular plaques and AGEs-induced HAECs. METTL3 promoted *Foxo1* mRNA m^6^A modification and increased FOXO1 expression in a YTHDC1-dependent manner, thereby accelerating atherosclerosis and HAECs autophagic flux blockade leading to apoptosis (Fig. 8). The results revealed that: (Ⅰ) The m^6^A modification levels were significantly elevated in diabetic carotid artery plaques and diabetic atherosclerotic mouse aortic plaques. Under AGEs stimulation, HAECs exhibited increased m^6^A modification of *Foxo1* mRNA, along with upregulated expression of the m^6^A “writer” METTL3 and the “reader” YTHDC1. (Ⅱ) METTL3 expression was markedly increased in endothelial cells of diabetic carotid plaques and atherosclerotic mouse aortas. Knockdown of METTL3 significantly reversed AGEs-induced elevation of *Foxo1* mRNA m^6^A modification in HAECs, thereby reducing autophagic flux blockade leading to apoptosis. Importantly, FOXO1 overexpression abolished these protective effects, indicating that METTL3 acts through FOXO1 to promote AGEs‑induced endothelial injury. (Ⅲ) YTHDC1 expression was significantly upregulated in endothelial cells of diabetic carotid plaques and atherosclerotic mouse aortas. Under AGEs stimulation, YTHDC1 promoted FOXO1 expression by specifically recognizing the 3’-UTR-3 site of *Foxo1* mRNA, and YTHDC1 knockdown effectively suppressed AGEs-induced FOXO1 upregulation in HAECs. (Ⅳ) In endothelial-specific METTL3 knockout diabetic atherosclerotic mice, METTL3 deletion significantly attenuated plaque formation, reduced the expression of both METTL3 and FOXO1, and ameliorated autophagic flux blockade and subsequent apoptosis. Therefore, the METTL3/YTHDC1 axis promotes endothelial autophagic apoptosis in diabetic atherosclerosis by mediating m^6^A modification of *Foxo1* mRNA, identifying this pathway as a promising therapeutic target.

M^6^A methylation contributes significantly to the pathophysiology of both diabetes and cardiovascular disorders. Accumulating evidence has demonstrated significantly elevated m^6^A levels in renal tissues of diabetic mice and lens specimens from diabetic cataract patients(Jiang et al. 2022, Yang et al. 2020). Notably, substantial increases in m^6^A methylation have been consistently observed in AGEs-exposed podocytes, ox-LDL-stimulated HAECs, and TNF-α-treated human umbilical vein endothelial cells (HUVECs)(Tang et al. 2024, Jian et al. 2020, Wu et al. 2025). Our findings further reveal significant upregulation of m^6^A modification in human diabetic carotid plaques, aortic lesions of diabetic mice, and AGEs-induced HAECs.

A growing body of evidence has established the critical involvement of m^6^A regulatory factors-methyltransferases (METTL3, METTL14, WTAP, and KIAA1429) in cardiovascular pathologies. Among which, METTL3 have been found in atherosclerosis pathogenesis through distinct molecular mechanisms: METTL3 suppression enhancing *Socs3* mRNA stability to mitigate high glucose-induced endothelial damage(Li et al. 2023); accelerating vascular smooth muscle cell dysfunction through YTHDF3-mediated m^6^A-dependent translation of *Mrtfa* mRNA(Dong et al. 2025); promoting cellular proliferation, migration and angiogenesis in ox-LDL-stimulated endothelial cells(Kobayashi et al. 2018); mediating oscillatory shear stress-induced endothelial m^6^A RNA methylation, inflammation and monocyte adhesion(Chien et al. 2021). In addition, METTL3 has been shown to facilitate type 2 diabetic vascular complications by upregulating EGR1 expression and promoting endothelial-to-mesenchymal transition(Tao et al. 2025). Our findings further revealed significant METTL3 upregulation in both diabetic vascular plaques and AGEs-induced HAECs, endothelial-specific METTL3 knockout markedly inhibited atherosclerotic plaque formation in diabetic mouse models, further substantiating METTL3 inhibition offering therapeutic potential for diabetes-associated atherosclerosis.

FOXO1 exhibits characteristic nuclear localization and serves as a master regulator in maintaining metabolic homeostasis, cell cycle progression, oxidative stress response, and immune modulation(Xing et al. 2018, Benchoula et al. 2021). Emerging evidence has demonstrated that FOXO1 knockdown ameliorates retinal DNA damage and vascular dysfunction in diabetic retinopathy(Sun et al. 2023), while FOXO1 promotes endothelial quiescence by antagonizing MYC, leading to coordinated reduction in endothelial cell proliferation and metabolic activity(Wilhelm et al. 2016). Substantial studies have established pivotal involvement of FOXO1 in atherosclerosis pathogenesis(Zhang et al. 2021, Zhang et al. 2023), with its knockdown enhancing plaque stability(Zhang et al. 2023) and endothelial-specific FOXO1 deletion significantly attenuating atherosclerotic plaque formation in mice(Tsuchiya et al. 2012). Our previous work revealed participation of FOXO1 in AGEs-induced autophagic apoptosis in HAECs(Zhang et al. 2019). The current study further revealed that AGEs upregulated METTL3 expression in HAECs. Knockdown of METTL3 significantly reversed AGEs-induced elevation of *Foxo1* mRNA m^6^A modification and FOXO1 expression, thereby reducing autophagic flux blockade and subsequent apoptosis in HAECs.

The m^6^A modification machinery operates through a coordinated “writer-reader-effector” axis where reader proteins (YTHDF/YTHDC/IGF2BP family members) recognize and execute the functional consequences of m^6^A marks installed by writer proteins, thereby regulating critical RNA metabolic processes including splicing, nuclear export, degradation and translation(Wang et al. 2015, Wang et al. 2014). Substantial evidence has implicated YTH domain-containing proteins in the pathogenesis of diabetes and its vascular complications through m^6^A-dependent regulatory mechanisms. Specifically, YTHDC1 downregulation impaired diabetic wound healing by promoting *SQSTM1* nuclear mRNA degradation and subsequent autophagy inhibition in keratinocytes(Liang et al. 2022), while YTHDC2 mediated vascular smooth muscle cell dysfunction under high-glucose conditions through regulation of m6A-modified transcripts(Yuan et al. 2021), and YTHDF1 contributed to atherogenesis in high-fat diet conditions by recognizing m^6^A sites on *Braf* mRNA to control its translational output(Li et al. 2023). Our findings demonstrated significant upregulation of YTHDC1 expression in both diabetic vascular plaques and AGEs-induced HAECs. Study in neuroblastoma revealed that YTHDC1 promoted *Foxo1* mRNA degradation via m^6^A recognition, thereby disrupting FOXO1/β-catenin interactions and activating oncogenic Wnt/β-catenin signaling(Wang et al. 2025). However, the potential mechanism of YTHDC1 in regulating FOXO1 expression and its function in the context of diabetic complications remains unexplored. Our results established that AGEs robustly enhanced YTHDC1 binding to specific m^6^A sites located within the 3′-UTR-3 region of *Foxo1* mRNA, and knockdown of YTHDC1 effectively attenuates AGEs-induced FOXO1 upregulation, demonstrating a novel regulatory paradigm in which the METTL3-m^6^A-YTHDC1 axis controls FOXO1 expression through epitranscriptomic mechanisms in diabetic vascular pathology.

While the METTL3/YTHDC1/FOXO1 axis were upregulated in diabetic atherosclerosis, several limitations must be considered for further study. First, the use of a chow diet in diabetic apoe^⁻/⁻^ mice produced relatively mild atherosclerotic lesions compared with western diet. However, the plaque burden sufficed to confirm the model’s validity for our mechanistic analyses. Second, our MeRIP-seq detected over 1,100 differentially methylated peaks in AGEs-treated endothelial cells, besides FOXO1, other m⁶A targets (e.g., Becn1, Atg12) may also participate in this process, which were warranted further investigation. Third, besides METTL3/YTHDC1, other “writers” and “readers” might also regulate m^6^A RNA modifications of *Foxo1*. For example, knockdown of METTL14 in macrophage enhanced m^6^A modification of *Foxo1* to mediate endothelial inflammation and promote plaque formation(Zheng et al. 2022). It is crucial to comprehensively evaluate the regulatory network of *Foxo1* m^6^A modification. Meanwhile, our study showed that endothelial-specific METTL3 knockout could markedly attenuate plaque formation in diabetic atherosclerotic mice. Whether this can be developed a clinical intervention strategy remains to be verified. The recent approval of STC-15, a first-in-class METTL3 small-molecule inhibitor, for a phase 1b/2 clinical study in cancer patients underscores the therapeutic potential of targeting the m^6^A modification pathway(Tang et al. 2025).

In summary, our study provided epigenetic evidence demonstrating that RNA-m^6^A modification were increased in diabetic vascular plaques and AGEs-treated HAECs, along with the upregulation of METTL3/YTHDC1 axis. METTL3 mediates m^6^A modification of *Foxo1* mRNA and upregulates FOXO1 expression in YTHDC1-dependent manner. Knockdown of METTL3 significantly reversed AGEs-induced elevation of *Foxo1* mRNA m^6^A modification in HAECs, thereby reducing autophagic apoptosis. METTL3 deletion significantly attenuated plaque formation in endothelial-specific METTL3 knockout diabetic atherosclerotic mice. Our findings highlight the critical role of METTL3/YTHDC1 axis and m^6^A methylation in diabetic atherosclerosis, which might be novel targets for intervention.

## Supplementary Information

Supplementary Material 1.

Supplementary Material 2.

## Data Availability

The data of this study are available from the corresponding authors on reasonable request.

## Abbreviations

AGEs: Advanced glycation end products
ACTB: Actin beta
apoe^−/−^: apolipoprotein E knockout
ATG14: Autophagy-related 14
ALKBH5: AlkB homolog 5, RNA demethylase
BCL2: BCL2 apoptosis regulator
Bafilomycin A1: Baf-A1
CD31: CD31 antigen
EC: Endothelial cell
FTO: FTO alpha-ketoglutarate dependent dioxygenase
FOXO1: Forkhead box O1
GAPDH: Glyceraldehyde-3-phosphate dehydrogenase
HAECs: Human aortic endothelial cells
HE: Hematoxylin-eosin
KIAA1429: vir like m^6^A methyltransferase associated
KEGG: Kyoto Encyclopedia of Genes and Genomes
MeRIP: Methylated RNA immunoprecipitation
m^6^A: N6-methyladenosine
MAP1LC3/LC3: Microtubule-associated protein 1 light chain 3
METTL14: Methyltransferase 14, N^6^-adenosine-methytransferase complex catalytic subunit
METTL3: Methyltransferase 3, N6-adenosine-methytransferase complex catalytic subunit
MUT: Mutant-type
RIP: RNA immunoprecipitation
SQSTM1: Sequestosome 1
siRNA: small interfering RNA
WT: Wild-type
WATP: WT1 associated protein
YTHDF: YTH N^6^-methyladenosine RNA binding protein F
YTHDC: YTH N6-methyladenosine RNA binding protein C
