# Supplementary material for: METTL3/YTHDC1 axis-mediated m6A modification of Foxo1 mRNA promote endothelial autophagic apoptosis in diabetic atherosclerosis
Source: Mol Med. 2026 Jul 3;32:159. doi: 10.1186/s10020-026-01543-z (PMC13602580; doi:10.1186/s10020-026-01543-z)
Supplement: Supplementary file 1 [file 10020_2026_1543_MOESM1_ESM.pdf]

Supplementary figures

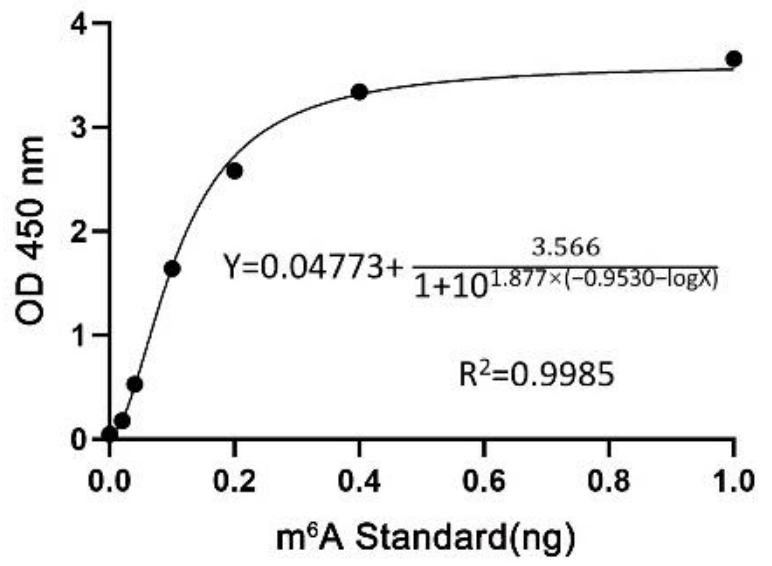

Figure S1. Standard curve for m^6A quantification. (A) Representative standard curve for quantitative detection of m^6A. Standard concentrations (ng) were plotted against the corresponding OD_{450nm} values. The curve was fitted with a 4-PL model, with the equation and R^2 value shown in the figure.

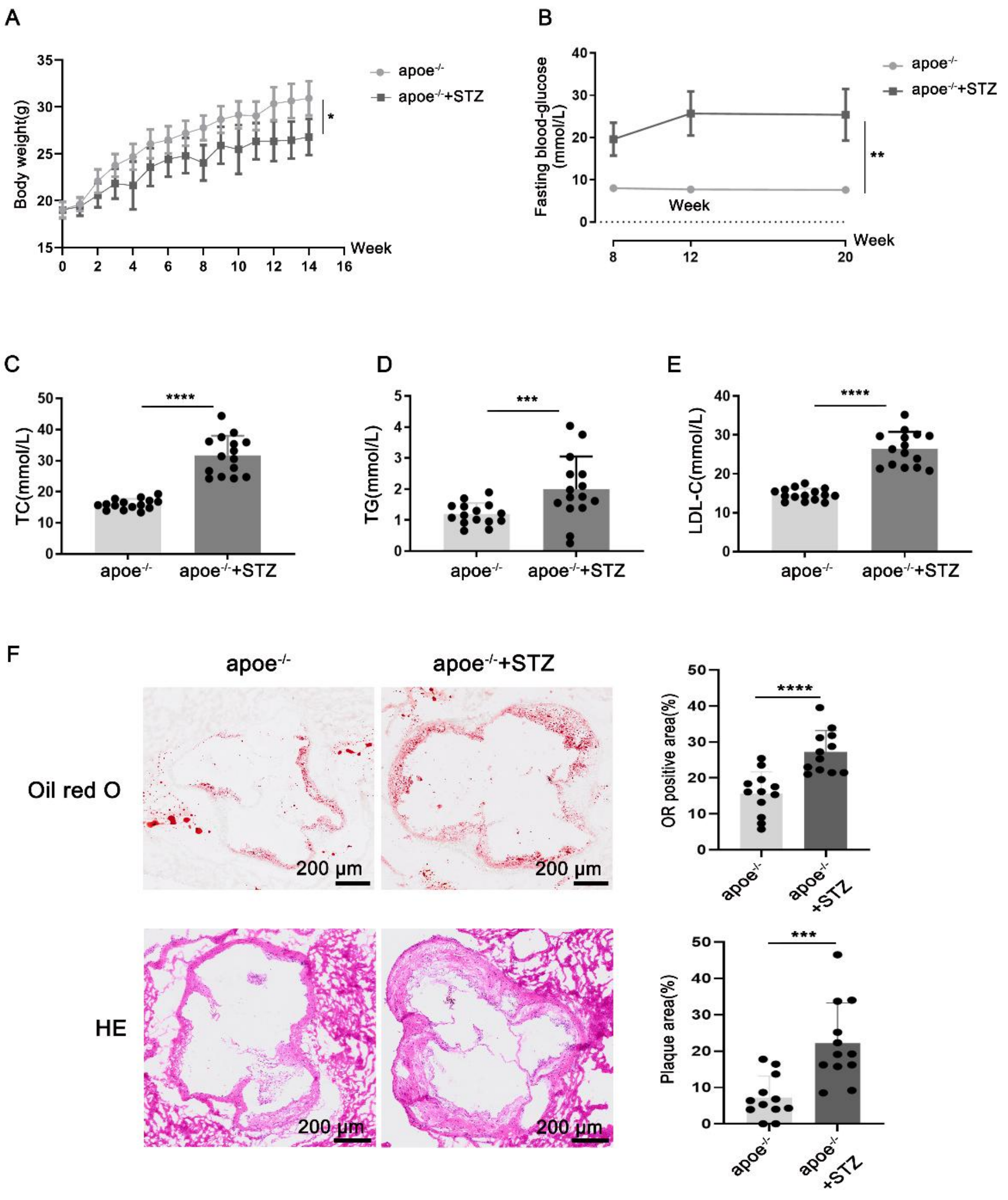

Figure S2. Comparison of body weight, blood glucose, lipid profiles, and atherosclerotic lesions between non-diabetic atherosclerotic mice and diabetic atherosclerotic mice. Changes in body weight (A), blood glucose (B), lipid profiles (C-E) in apoe$^{-/-}$ and apoe$^{-/-}$+STZ mice. n=15. (F) Representative images and quantitative analysis of Oil Red O staining, HE staining in the aortic tissues of apoe$^{-/-}$ and apoe$^{-/-}$+STZ mice. Scale bar=200 μm. n=12. Data are presented as mean ± SD, and analyzed by Student's *t*-test in all. $^{*}P < 0.05$, $^{**}P < 0.01$, $^{***}P < 0.001$, $^{****}P < 0.0001$.

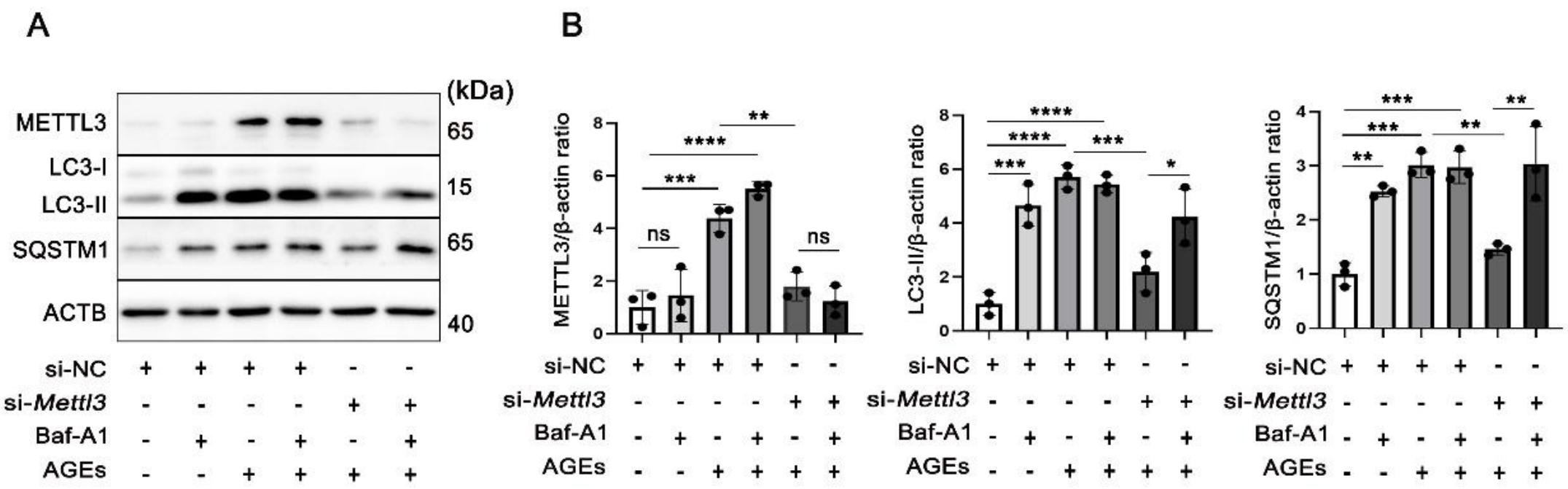

Figure S3. Treatment with Baf-A1 confirmed that METTL3 silencing restores autophagic flux upon AGEs stimulation. (A-B) Western blot analysis and quantification of METTL3, LC3-II, and SQSTM1 protein levels in HAECs subjected to si-NC or si-*Mettl3* transfection, with or without AGEs and/or Baf-A1 treatment. n = 3. Data are presented as mean ± SD and were analyzed by one-way ANOVA followed by Tukey's post hoc test. n = 3 independent biological replicates. $^{*}P < 0.05$, $^{**}P < 0.01$, $^{***}P < 0.001$, $^{****}P < 0.0001$, ns, not significant.

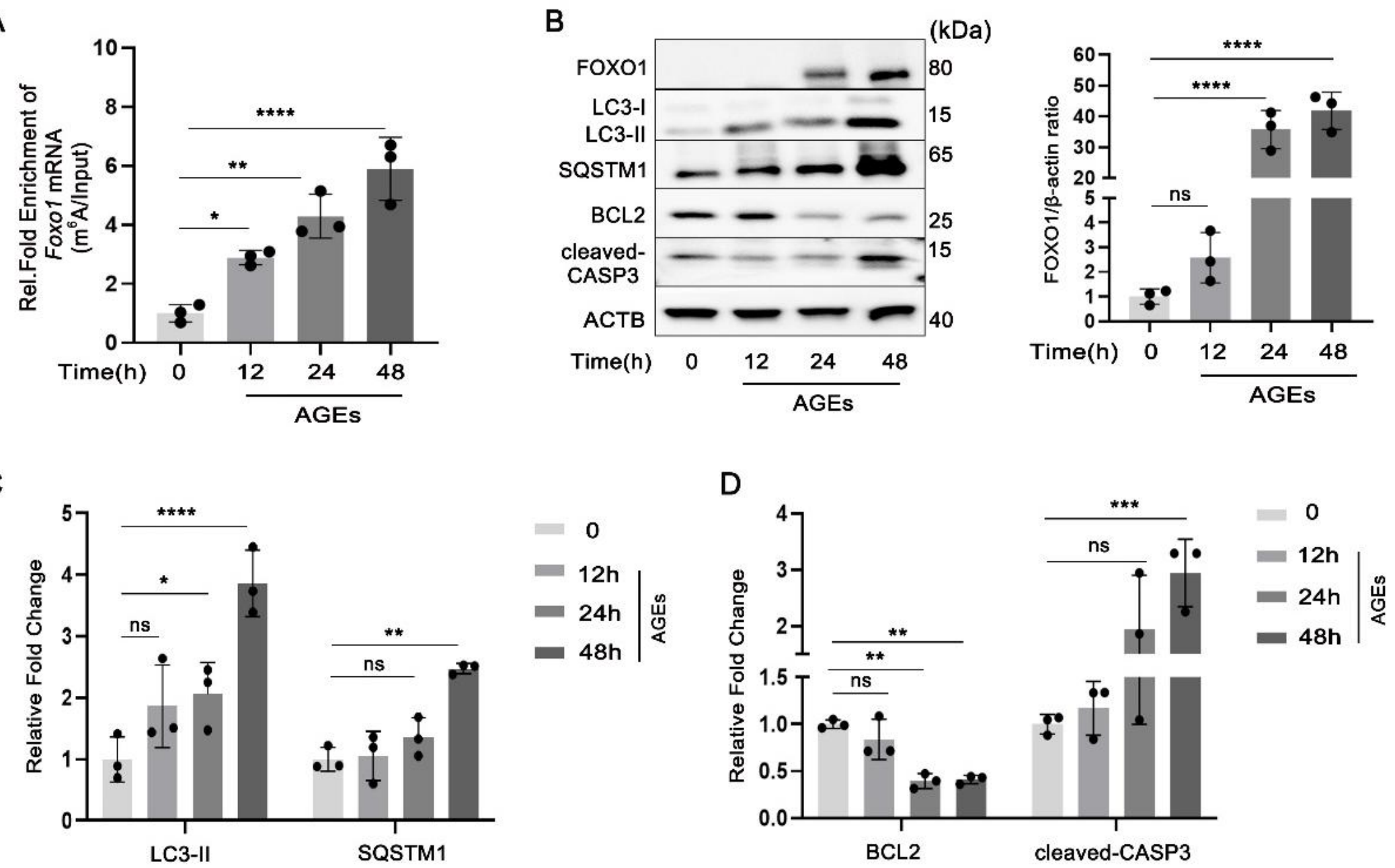

Figure S4. Temporal relationship among m⁶A modification, autophagy, and apoptosis. (A) MeRIP-qPCR analysis showing the relative enrichment of *Foxo1* mRNA pulled down by an m⁶A-specific antibody in HAECs treated with AGEs for 0, 12, 24, or 48 h,

with *Foxo1* abundance quantified by qRT-PCR. n = 3. (B) Representative western blot images and quantification of FOXO1 protein levels in HAECs treated with AGEs for the indicated time points. n = 3. (C–D) Representative western blot images and quantification of autophagy-related (LC3-II, SQSTM1) and apoptosis-related (BCL2, cleaved-CASP3) protein levels in HAECs treated with AGEs for the indicated time points. n = 3. Data are presented as mean ± SD and were analyzed by one-way ANOVA followed by Tukey's post hoc test. n = 3 independent biological replicates. $^{*}P < 0.05$, $^{**}P < 0.01$, $^{***}P < 0.001$, $^{****}P < 0.0001$, ns, not significant.

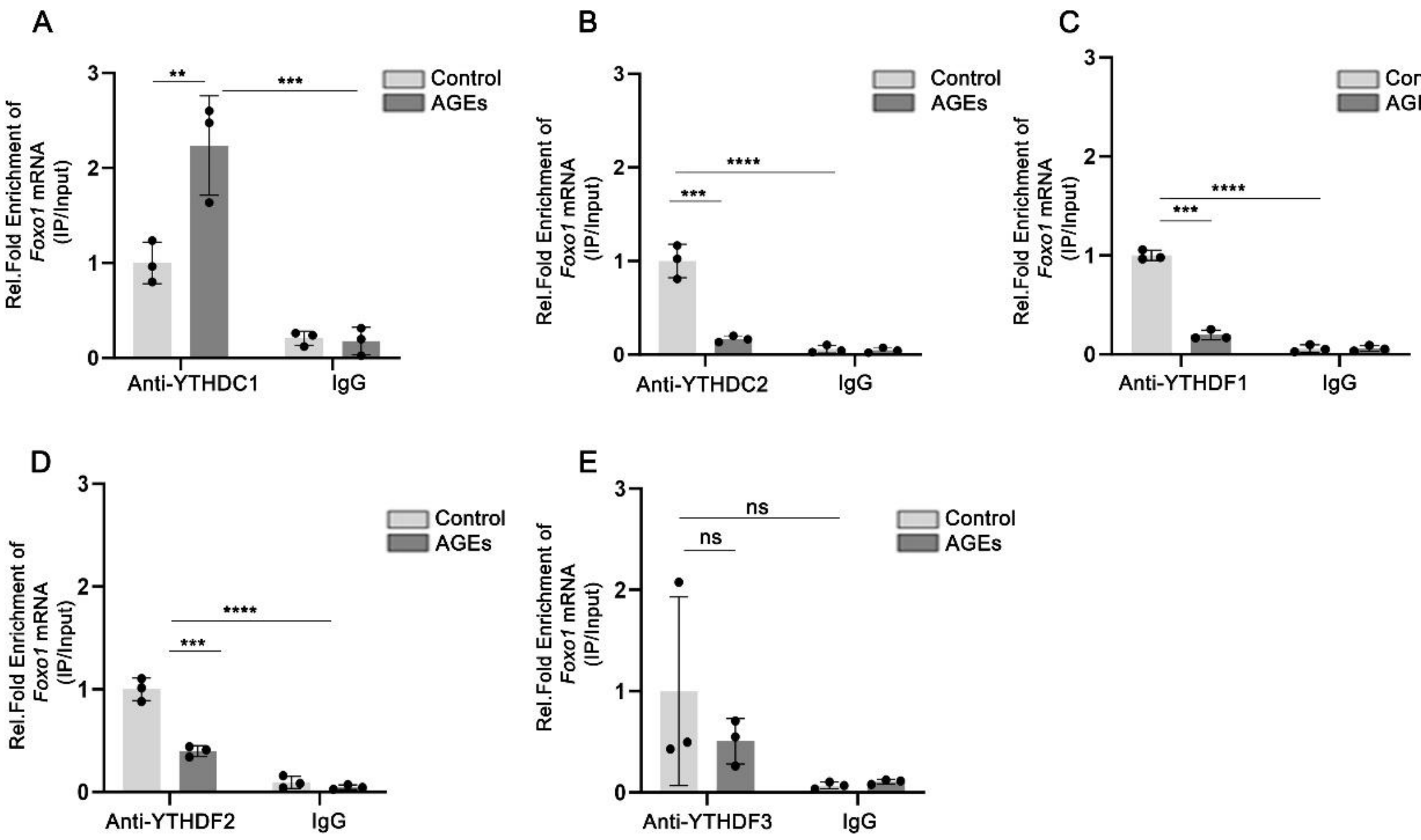

Figure S5. Individual RIP-qPCR analysis of YTHDC1, YTHDC2, YTHDF1, YTHDF2 and YTHDF3 binding to *Foxo1* mRNA with IgG negative control. (A-E) Separate RIP-qPCR results for YTHDC1, YTHDC2, YTHDF1, YTHDF2 and YTHDF3 respectively. Relative *Foxo1* mRNA enrichment was normalized to input RNA (IP/Input ratio). IgG served as negative control for non-specific immunoprecipitation. Data are presented as mean ± SD and analyzed by two-way ANOVA followed by Tukey's post hoc test. $^{*}P <$

0.05, $^{**}P < 0.01$, $^{***}P < 0.001$, $^{****}P < 0.0001$, ns, not significant.

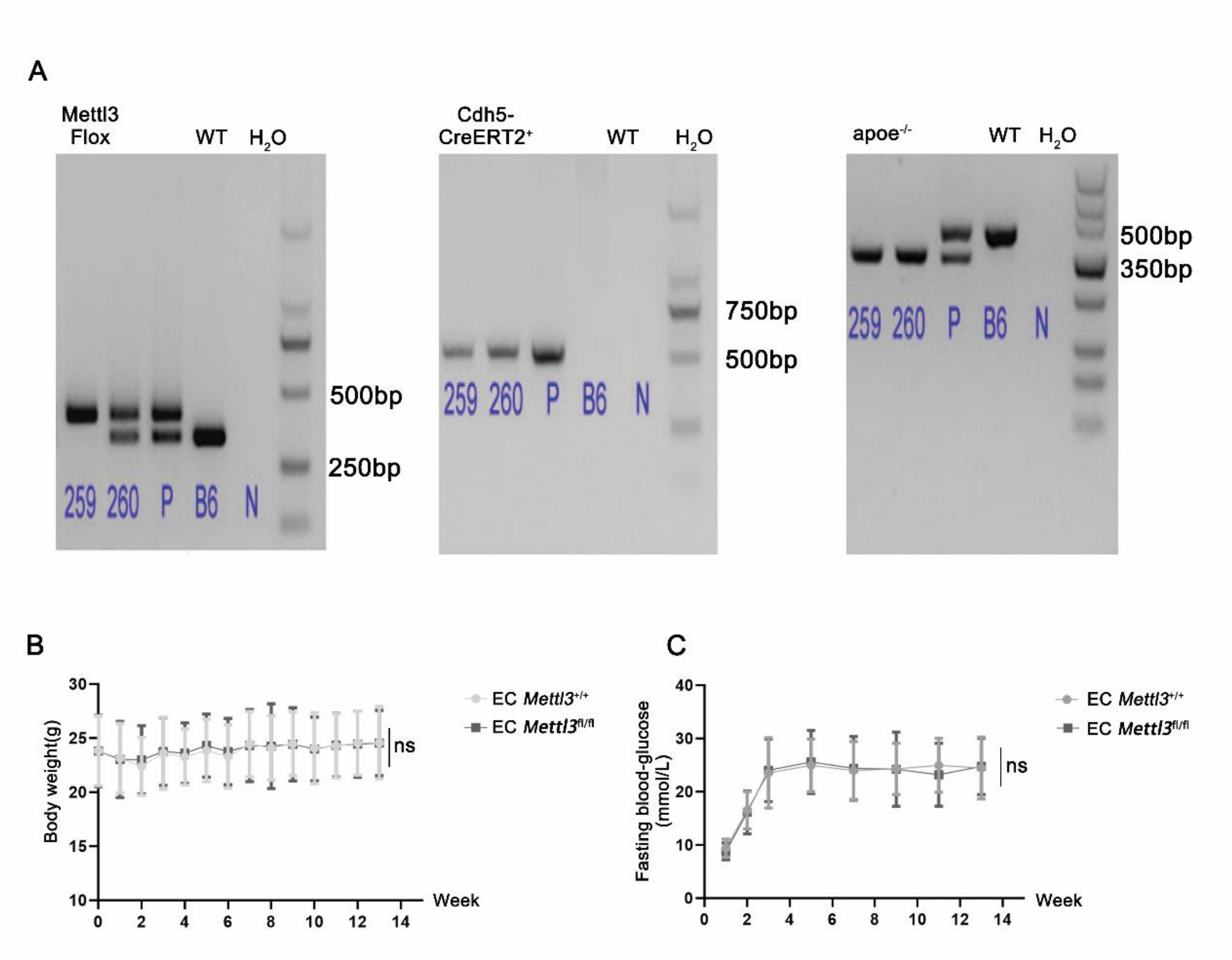

Figure S6. Genotype confirmation and assessment of body weight and blood glucose levels in endothelial-specific *Mettl3* knockout mice. (A) Representative genotyping PCR results for *Mettl3*$^{fl/fl}$, Cdh5-CreERT2^{+} and apoe$^{-/-}$ mice. Changes in body weight (B) and blood glucose (C) in EC *Mettl3*$^{+/+}$ and EC *Mettl3*$^{fl/fl}$ mice. n=20-22. Data are presented as mean ± SD, and analyzed by Student's *t*-test in (B and C). ns, not significant.

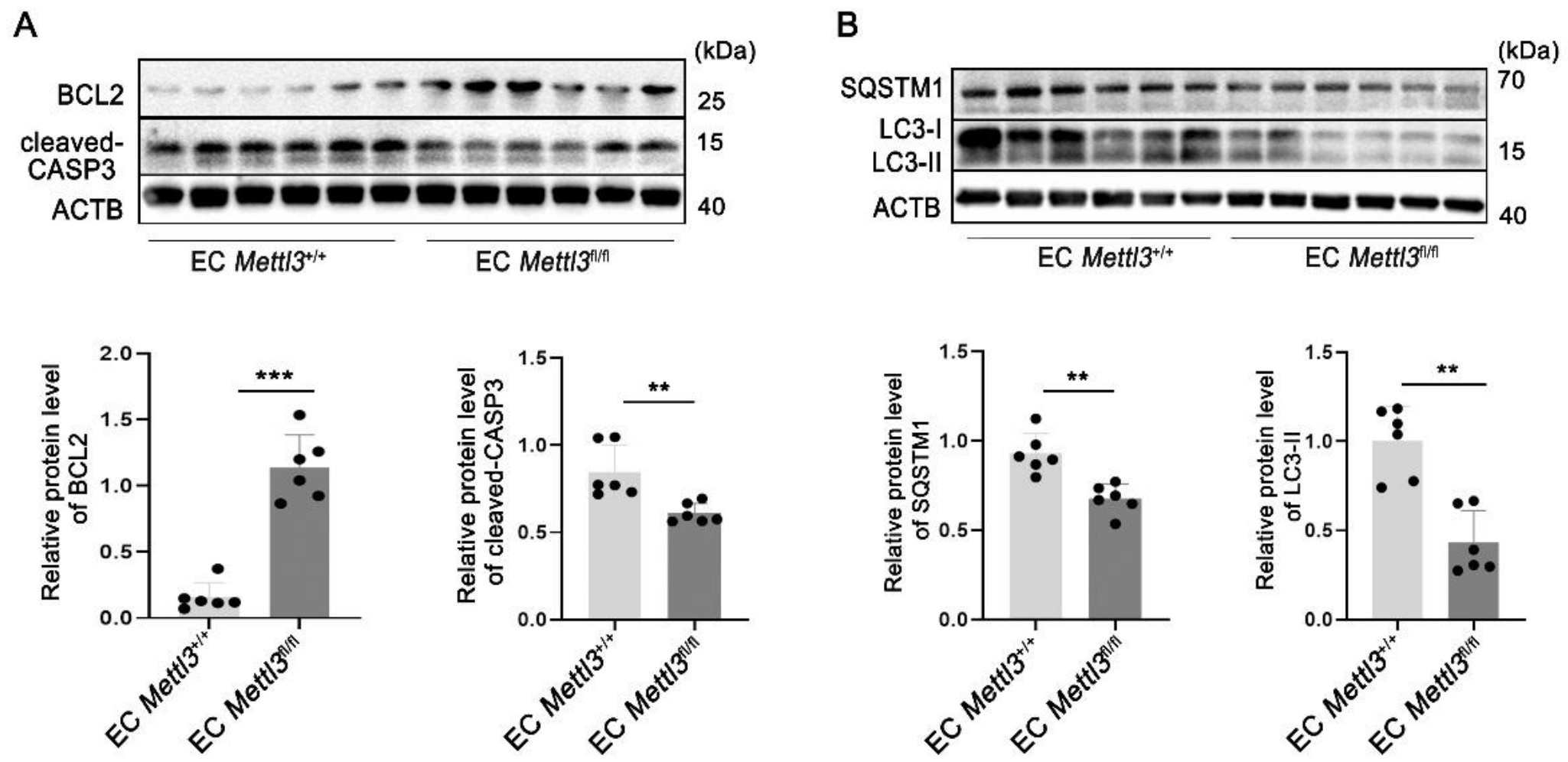

Figure S7. Endothelial cell-specific knockout of METTL3 significantly ameliorates autophagic apoptosis in diabetic atherosclerosis mice. Representative Western blot images and quantitative analysis of BCL2 and cleaved-CASP3 (A) SQSTM1 and LC3-II (B) Expression in aortic plaques of STZ-treated EC *Mettl3*$^{+/+}$-apoe$^{-/-}$ and EC *Mettl3*$^{fl/fl}$-apoe$^{-/-}$ mice. n=6. Data are presented as mean ± SD, and analyzed by Student's *t*-test in (A and B). $^{*}P < 0.05$, $^{**}P < 0.01$, $^{***}P < 0.001$, $^{****}P < 0.0001$.
