# Supplementary material for: METTL3/YTHDC1 axis-mediated m6A modification of Foxo1 mRNA promote endothelial autophagic apoptosis in diabetic atherosclerosis
Source: Mol Med. 2026 Jul 3;32:159. doi: 10.1186/s10020-026-01543-z (PMC13602580; doi:10.1186/s10020-026-01543-z)
Supplement: Supplementary file 2 [file 10020_2026_1543_MOESM2_ESM.pdf]

Supplementary tables

Table S1 Clinical characteristics.

	**No diabetes (n=8)**	**Diabetes (n=8)**
**Age (Years)**	65.875±5.87	71.75±2.05
**Sex**	Male (n=6), Female(n=2)	Male (n=7), Female(n=1)
**Fasting blood glucose （mmol/L）**	5.13±0.48	9.12±3.48[a]
**Haemoglobin A1c (%)**	5.54±0.55	7.96±1.24[a]
**Total cholesterol (mmol/L)**	3.68±0.88	3.44±1.26
**HDL cholesterol (mmo/L)**	1.29±0.28	0.93±0.18
**LDL cholesterol (mmoVL)**	1.86±0.62	1.69±0.86
**Triglycerides (mmoVL)**	1.08±0.60	1.82±0.55
**Systolic blood pressure (mmHg)**	136.38±6.21	140.63±8.93
**Diastolic blood pressure (mmHg)**	73.75±7.17	74.88±7.86

Data are presented as mean ± SD. HDL, high-density lipoprotein, LDL, low-density lipoprotein. [a]$p < 0.05$ by unpaired t-test.

Table S2 Raw OD_{450nm} values of standards and individual samples for m^6A quantification (sample RNA=200ng).

m^6A stardard OD_{450nm}	m^6A stardard(ng)	no diabetes	no diabetes OD_{450nm}		diabetes	diabetes OD_{450nm}	
0.048	0	Sample1	0.59	0.595	Sample1	0.91	0.78
0.177	0.02		0.6			0.65	
0.529	0.04	Sample2	0.54	0.53	Sample2	0.95	1.03
1.643	0.1		0.52			1.11	
2.583	0.2	Sample3	0.5	0.49	Sample3	0.65	0.68
3.342	0.4		0.48			0.71	
3.657	1						

Table S3 Characteristics of $apoe^{-/-}$ and $apoe^{-/-}$+STZ mice with diabetic atherosclerosis induction.

Groups	$apoe^{-/-}$ (n=15)	$apoe^{-/-}$+STZ (n=15)
**Age (Week)**	6	6
**Weight before (g)**	19±0.80	19±0.90
**Weight after (g)**	30.80±1.89	25.9±2.02
**Fasting blood glucose （mmol/L）**	7.60±0.77	25.40±6.12
**TC (mmol/L)**	16.01±1.67	31.76±6.28
**TG (mmol/L)**	1.20±0.35	2.00±1.05
**LDL-C (mmol/L)**	14.72±1.52	26.42±4.42

Data are presented as the mean ± SD. TC, total cholesterol; TG, triglyceride.

Table S4 Atherosclerotic lesion quantification in the chow-fed $apoe^{-/-}$ and $apoe^{-/-}$ +STZ.

Groups	$apoe^{-/-}$ (n=3)	$apoe^{-/-}$+STZ (n=3)
**Plaque Burden (%, mean ± SD)**	13.14±2.05	25.15±7.16[a]

Data are presented as the mean ± SD. [a]$p < 0.05$ by unpaired t-test.

Table S5 Characteristics of EC *Mettl3*$^{+/+}$ and EC *Mettl3*$^{fl/fl}$ mice with diabetic atherosclerosis induction.

Groups	EC *Mettl3*$^{+/+}$ (n=22)	EC *Mettl3*$^{fl/fl}$ (n=20)
**Age (Week)**	8	8
**Weight before (g)**	24.90±3.3	24.80±3.5
**Weight after (g)**	24.60±3.36	24.6±3.02
**Fasting blood glucose （mmol/L）**	24.72±4.99	23.99±5.27

For induction of diabetic atherosclerosis, tamoxifen-treated EC *Mettl3*$^{+/+}$ and EC *Mettl3*$^{fl/fl}$ mice were injected with STZ (55 mg/kg) for 5 days and fed a standard diet until 20 weeks of age. The data are presented as the mean ± SD.
